# Osteocytes Produces RANKL Via Wnt-TGFβ Signaling Axis for Osteoclastogenesis

**DOI:** 10.7150/ijbs.117481

**Published:** 2025-09-12

**Authors:** Yujiao Liu, Lizhou Zhao, Molin Li, Weimin Gong, Xiaofang Wang, Yu Cheng, Ying Zhang, Pengtao Wang, Yisheng Luo, Yining Zhang, Yufei Shao, Makoto Mark Taketo, Teresita Bellido, Gaohai Shao, Xing Liu, Xiaolin Tu

**Affiliations:** 1 Laboratory of Skeletal Development and Regeneration, Key Laboratory of Clinical Laboratory Diagnostics (Ministry of Education), College of Laboratory Medicine, Chongqing Medical University, Chongqing 400016, China.; 2 Department of Orthopedics, Affiliated Yongchuan Hospital of Chongqing Medical University, Chongqing 402160, China.; 3 Department of Nursing, Affiliated University City Hospital of Chongqing Medical University, Chongqing 401331, China.; 4 Department of Orthopedics, Ministry of Education Key Laboratory of Child Development and Disorders, National Clinical Research Center for Child Health and Disorders, Children's Hospital of Chongqing Medical University, Chongqing, 400014, China.; 5 Department of Pharmacology, Graduate School of Medicine, Kyoto University, Kyoto, 606-8501, Japan.; 6 Department of Physiology and Cell Biology, University of Arkansas for Medical Sciences, Little Rock, AR 72223, USA.; 7 Department of Anatomy, Cell Biology and Physiology, Indiana University School of Medicine, Indianapolis, IN, 46202, USA.

**Keywords:** Osteocyte, Wnt/β-catenin, osteoclastogenesis, TGFβ, RANKL

## Abstract

Osteocytes are derived from osteoblasts in the mineralized matrix and are the main source of RANKL required for osteoclastogenesis. We initially found osteocytes as central target cells for Wnt/β-catenin signaling that increases RANKL expression and bone resorption in mice. However, how RANKL is regulated remains unclear. Here, we demonstrated its role and molecular mechanisms using primary osteocytes isolated from long bones. Osteocyte transcriptome sequencing revealed the most associated osteoclast differentiation in KEGG pathways with upregulated expression of *Tgfb1/2*. *In vivo* data highlight the specificity of osteocytic Wnt, rather than osteoblastic Wnt, in regulating TGFβ signaling. Activation/inactivation of osteocytic TGFβ signaling stringently promotes/inhibits RANKL expression and osteoclast differentiation in dose- and time-dependent manners. Wnt signaling increases RANKL expression through TGFβ signaling via the physical interaction of its transcription factor Smad4 with the RANKL promoter region. Mice with disrupted TGFβ signaling in osteocytes recapitulate defective osteoclastogenesis and reduced RANKL expression in osteocytes. Thus, osteocytes mediate bone resorption via Wnt-TGFβ signaling axis.

## Introduction

Osteoclasts are multinucleated giant cells with bone resorptive function, formed from monocyte/macrophage fusion in a variety of ways^1^. They degrade bone by secreting acids and proteolytic enzymes that dissolve collagen and other matrix proteins^2^, ^3^. Their physiological differentiation requires the receptor activator of nuclear factor-κB ligand (RANKL), a member of the TNF superfamily^2^, which binds to the membranous receptor activator of nuclear factor-κB (Rank) of osteoclast precursors and regulates osteoclast differentiation (OcD) by modulating the NF-κB pathway, leading to the production and activation of the key transcription factor NFATc1 for OcD^4^-^6^. RANKL is primarily produced by osteocytes^7^-^9^, and combines with the macrophage colony-stimulating factor (MCSF) that proliferates osteoclast precursors, to induce the formation of osteoclasts capable of bone resorption*.* Its decoy receptor, osteoprotegerin (Opg), which is also secreted by osteocytes and osteoblasts, inhibits OcD by blocking the binding of RANKL to Rank. Deficiency of Opg in both mice and humans leads to an increase in OcD, which can lead to severe osteoporosis^4^. Therefore, the RANKL/Opg ratio, the relative amount of RANKL and Opg is the indicator of the magnitude of OcD in physiological condition.

There has been controversy regarding the source of RANKL, and in the past, it was thought that: osteoblasts and bone marrow stromal cells, as well as CXC chemokine ligand 12-rich reticulocytes, chondrocytes, and lymphocytes produce RANKL to support osteoclastogenesis^10^. However, it has been proposed that the major source of RANKL is osteocytes, which express higher levels of RANKL than the cell types mentioned above. RANKL-neutralizing antibodies have been used for many years in clinical anti-osteoporosis therapies but, regardless of cell type, there is no consensus on how RANKL is generated, let alone the discovery of its transcription factors.

Wnt/β-catenin plays major roles on bone formation and resorption^11^, however, its regulation of bone resorption is typically based on its role in osteoblasts, specifically, it reduces the RANKL/Opg ratio by upregulating the expression of Wnt-targeted gene *Opg*, which reduces OcD to increase bone mass^12^, ^13^. Subsequent studies have also found that Wnt ligands Wnt3a^14^-^16^ and Wnt16^17^ indirectly inhibit osteoclast formation by increasing Opg expression in osteoblastic cells through canonical Wnt signaling. On the other hand, osteoblast-derived Wnt16^17^ and Wnt4^18^ can inhibit the NF-κB pathway to decrease the expression of NFATc1, thereby directly inhibiting OcD to prevent bone loss in aging and osteoporosis patients. In addition, Wnt5a secreted by osteoblasts promotes OcD through the non-canonical Wnt pathway via the receptor Ror2 in osteoclast precursors^19^. However, in either case, osteoblasts account for only 5% of the total number of bone cells in the skeleton, which poses a great challenge for the application of targeted osteoblasts.

Osteoblasts secrete collagen to wrap around themselves and then develop into terminally differentiated osteocytes, which account for more than 90% of the total number of cells in bone. Thus, when we initially found that degenerative osteocytes, which are otherwise poor metabolizers in their own right, produce intense bone formation when activating Wnt/β-catenin signaling, and also possess unexpectedly vigorous bone resorption^20^. These findings have been commented on as an important physiological function of the skeleton^21^, ^22^ that hides a secret of the skeleton's ability to regulate its own homeostasis. This unexpected phenomenon attracted our attention: the regulation of bone resorption by osteocytic Wnt is completely opposite to that of osteoblastic Wnt. Assays at the molecular level showed increased levels of Opg expression in bone, but the mRNA and protein levels of RANKL increased even more, resulting in a net increase in the RANKL/Opg ratio. Therefore, we hypothesized that osteocytes regulate RANKL expression to promote OcD upon stimulation by Wnt signaling.

In this study, primary osteocytes with dominantly active β-catenin (daCO) were isolated and cultured from the tibiae and femurs of Wnt mice (daβcat^Ot^)^20^ and their *ex vivo* and *in vivo* cellular biological functions were investigated. In contrast to the negative effect of Wnt on TGFβ signaling in osteoblasts, we emphasized the specificity of Wnt signaling in regulating TGFβ signaling in osteocytes. Wnt signaling enhances the expression of RANKL through TGFβ signaling, which is activated by the signaling transcription factor Smad4 through physical interactions that activate the transcriptional activity of RANKL, thereby increasing the RANKL/Opg ratio that promotes OcD and bone resorption. This regulatory role was genetically evidenced after disruption of osteocytic TGFβ signaling in mice. Thus, these data enrich the knowledge of developmental biology that Wnt signaling has a second function in regulating OcD: osteocytic Wnt promotes RANKL gene transcription, reveals that bone cells have different functions at different stages of bone development, indicating the spatiotemporal nature of Wnt signaling function. Based on the large number of osteocytes and their long-lived lifespan (more than ten years in humans), osteocytes are expected to be a new therapeutic target.

## Materials and Methods

### Reagent

Wnt agonist CHIR99021 (C91)^23^, TGFβ signaling-activator SRI-011381 (SRI) and TGFβ signaling inhibitor ITD-1 were all purchased from MedChemExpress (Monmouth Junction, NJ, USA), 1,25-dihydroxyvitamin D_3_ (VitD_3_), prostaglandin E_2_ (PGE_2_), RANKL and MCSF recombinant proteins from PeproTech (Rocky Hill, New Jersey, USA). The method for collecting Wnt3a cell culture supernatant used to activate classical Wnt signaling was performed as previously described^24^. Tgfb1 and Tgfb2 recombinant protein were purchased from TargetMol (Boston, Massachusetts, USA), and dissolved in HCl to the specified concentration according to the manufacturer's instructions.

### Mouse generation

The DMP1-8kb-Cre^25^, Catnb^lox(ex3)^^26^, Col1α1-Cre/ER^T2^^27^ and TGFβRII floxed^28^ mouse strains are as previously described, were all on a C57BL/6 background. The Wnt mouse with dominant active Wnt/β-catenin signaling in osteocytes (daβcat^Ot^) were generated by crossing DMP1-8kb-Cre mice with Catnb^lox(ex3)^ mice, and the mice with osteocyte-specific inactivation of TGFβ signaling (TGFβRII^ΔOt^) by crossing DMP1-8kb-Cre mice with TGFβRII^f/f^ mice. The mouse with dominant active Wnt/β-catenin signaling in osteoblasts (i-daβcat^Ob^) were generated by crossing Col1a1-Cre/ER^T2^ mice with Catnb^lox(ex3)^ mice, and received Tamoxifen (Sigma-Aldrich, St. Louis, MO, USA) (130 µg/g, daily) for 5 days at 6 weeks of age to induce activation of Cre. C57BL/6 wild type (WT) mice were provided by the Experimental Animal Center of Chongqing Medical University. All experimental animals were not distinguished by sex, as the results were consistent in both genders. All animal procedures were approved by the Animal Care and Use Committee of Chongqing Medical University (approval number: IACUC-CQMU-2025-0094).

### Preparation of bone marrow monocytes and macrophages (BMM) and primary osteocytes

Isolation of BMM was performed as previously reported^29^, bone marrow cells were flushed out of the long bones of 2~4-month-old C57BL/6 mice and cultured in tissue culture dish overnight to remove adherent cells. None adherent cells were transferred to new dishes and cultured in α-MEM (Gibco, Grand Island, NY, USA) containing 10% fetal bovine serum (FBS, Wisent, Montreal, Canada) and 30 ng/ml MCSF recombinant protein for another 3 days, and adherent cells are BMM. Osteocyte isolation was performed exactly as previously described^30^, the long bones of mice were cut into small pieces after flushing out bone marrow, and subjected to alternating digestion with 1 mg/mL type I collagenase (Sigma-Aldrich, St. Louis, MO, USA) (15 min per cycle) and 4 mM EDTA (Sigma-Aldrich, St. Louis, MO, USA) (5 min per cycle) in a 37°C shaker, repeated for three cycles. The bone fragments were then cultured in α-MEM medium supplemented with 10% FBS and 1% penicillin/streptomycin under a humidified atmosphere of 37°C and 5% CO₂, allowing primary osteocytes to be released from the bone fragments.

### Osteoclast differentiation assay

To examine the effects of osteocytes on OcD, we co-cultured osteocytes and BMM as previously reported^31^. Briefly, primary osteocytes were seeded on 24-well plates at 1x10^4^/well overnight, BMM were added in at 4 × 10^4^/well to be co-cultured with the osteocytes until the formation of osteoclasts. Culture medium includes growth medium (α-MEM containing 10% FBS), OcD medium (the growth medium containing 50 ng/ml RANKL and 30 ng/ml MCSF), and osteoclast supplementary medium (the growth medium containing 50 nM VitD_3_ and 1 nM PGE_2_)^7^, ^32^. These 3 media were used as indicated in the results. The culture medium was changed half every 3 days. After 3-9 days, the cells were observed for the formation of multinucleated giant cells and harvested immediately for RNA extraction or TRAP staining.

### Tartrate-resistant acid phosphatase (TRAP) staining

For cell culture, TRAP staining was performed as reported^30^. Briefly, the cells were fixed with 10% buffered formalin for 5 min and air dried completely, then stained with the staining solution according to the instruction of the TRAP staining kit (Sigma-Aldrich, St. Louis, MO, USA). TRAP positive multinuclear cells (≥3 nuclei) and cells (≤2 nuclei) were counted as osteoclasts and osteoclast precursors per unit area under microscope. Tissue TRAP staining was carried out as described^30^. Shortly, paraffin sections of the femurs were rehydrated through graded ethanols to distilled water, and placed in 37°C pre-warmed TRAP Staining Solution Mix containing 0.6 mg/ml Fast Red Violet LB Salt (Sigma-Aldrich, St. Louis, MO, USA) and 0.1 mg/ml Naphthol AS-BI Phosphate (Sigma-Aldrich, St. Louis, MO, USA) for 30 min, and counterstained with 0.02% Fast Green (Sigma-Aldrich, St. Louis, MO, USA) for 30 sec. Osteoclast number was normalized to bone perimeter as N.Oc/B.Pm and the eroded surface of bone per osteoclast as Oc.s/N.Oc was calculated using OsteoMeasure High Resolution Digital Video System (Atlanta, GA, USA). Take photos through the optical microscope (Olympus BX53, Tokyo, Japan) with type of the objective lenses: UPlanFLN, magnification: 10X/20X, and numerical aperture: 0.3/0.5.

### Cell proliferation activity assay

In order to test the function of daCO on BMM proliferation activity, the Cell Counting Kit-8 kit (CCK- 8, Beyotime, China) was conducted as reported^23^. The osteocytes and BMM were co-cultured in 96-well plates for 1, 3 and 5 days, 10 μL CCK-8 solution were added to each well followed by 2 h incubation, and measured absorbance at 450 nm by a microplate reader (Thermo Fisher Scientific, Carlsbad, CA, USA).

### Enzyme linked immunosorbent assay (ELISA) for RANKL and Opg

RANKL and Opg were detected in the culture medium by ELISA. Briefly, the medium of WTO or daCO culture was centrifuged at 1,200xg for 5 min to remove cellular debris. The RANKL and Opg concentration in supernatants were measured using ELISA kits (Ruixin Biotech, Quanzhou, China) according to the manufacturer's instructions. Absorbance at 450 nm was measured by a Microplate Reader.

### Bone resorption pit assay

Osteoclast activity was examined as previously described^33^. Briefly, bone slices were prepared from the diaphysis of a bovine femur. The fresh bovine femur was cut into small pieces with a chainsaw, then ground into 100 μM-thick slices with a grinder and sandpaper, and disinfected in 75% alcohol for 2 h and exposed under UV light for 1 h each side. Bone slices were soaked in PBS in 37°C overnight and then placed in a 48-well plate (1 slice/well), and osteocytes (5 × 10^3^/well) and BMM (2 × 10^4^/well) were added in and cultured with half medium change every 3 days. Osteoclast formation was observed under microscope within 5~11 days.

By harvest, bone slices were fixed with PBS-buffered 10% formalin for 5 min, stained with actin ring staining kit (YF 488-Phalloidin, UElandy, Suzhou, China) and TRAP-staining kit, and TRAP^+^ cells were counted under an optical microscope (Leica Dmi8, Wetzlar, Germany) with type of the objective lenses: FLUOTAR, magnification: 10X/20X, and numerical aperture: 0.32/0.55.

After the cells on bone slices were removed away by wet cotton, the bone resorption pit area was stained with 0.1% toluidine blue containing 1% sodium borate for 1 min. After rinsing, the bone slices were air dried and examined for the ratio of bone resorption pit area to bone area by OsteoMeasure High Resolution Digital Video System.

### RNA extraction and gene expression analysis

The total RNA of cells and bone tissue extraction and quantitative real-time PCR (qPCR) were performed as reported^20^, ^34^. Briefly, total RNA was extracted from the cells treated by Trizol (Thermo Fisher Scientific, Massachusetts, USA). The cDNA was synthesized by using a high-capacity cDNA reverse transcription kit (Accurate Biology, Hunan, China) and used as the templates for qPCR with primer sets (Supplementary information, Table S1). Relative mRNA expression levels were normalized to the housekeeping gene ribosomal protein S2 (ChoB) for osteocytes or GAPDH for formed osteoclasts by using the ΔCt method^20^.

### Western blotting

According to the method previously reported^23^, cells were lyzed in RIPA buffer (Solarbio, Beijing, China) with 1% protease and 1% phosphatase cocktail inhibitors (Roche, Basel, Switzerland) on ice for 30 min; the protein supernatant was collected after centrifugation (12,000xg, 4℃, 5 min). 30 μg total protein were separated by 10% SDS-PAGE gel and transferred onto polyvinylidene fluoride membrane (Millipore, MA, USA), and blotted with antibodies specific to p-Smad2/3 (1:500, WL02305, Wanleibio, Shenyang, China), RANKL (1:1500, WL00285, Wanleibio, Shenyang, China), Opg (1:1500, WL02078, Wanleibio, Shenyang, China) and GAPDH (1:3,000, CST#97166, Cell Signaling Technology, MA, USA), and then, the membranes were incubated with horseradish peroxidase labeled secondary anti-mouse (Cell Signaling Technology, MA, USA) or anti-rabbit IgG (H + L) (Beyotime, Shanghai, China). Signals were detected with chemiluminescence blotting reagents (Beyotime, Shanghai, China) using a Bio-Rad XRS chemiluminescence detection system (Bio-Rad, Hercules, CA, USA).

### siRNA knockdown of RANKL

siRNA knockdown was performed as previously described^35^. Primary osteocytes were grown to 60~80% confluence and changed medium with antibiotic-free α-MEM with 1% FBS before transfection. Transfection supermix of siRNAs was made according to the manufacturers' instructions, i.e., 1.5 μL of 20 μM of siRNA was mixed with 1.5 μL advanced transfection reagent (ZETA life, Menlo Park, CA, USA) per 500 μL transfection solution, incubated for 10 min, and then added in each well. 48 h later, cells were harvested for RNA extraction for qPCR detection of *RANKL* and *Opg* expression, or subsequent co-culture with BMM for OcD. Details of siRNA sequences are listed in Supplementary information, Table S2.

### High-throughput sequencing data analysis

Primary osteocytes extracted from mouse femurs were subjected to RNA extraction for high-throughput sequencing analysis (RNA-seq) using the NovaSeq 6000 (Magorbio, Shanghai, China). The raw data were analyzed using the ggplot2 package in R Programming Language. The data from each group were standardized to the same baseline using log2+1 normalization to more accurately reflect biological differences. Principal Component Analysis (PCA) was employed to assess the ability of the dataset to reflect within-group variation. Differentially expressed genes (P<0.05, Fold Change>1.5) were visualized with volcano plots and Venn diagrams. Pathway enrichment analysis was conducted using the Kyoto Encyclopedia of Genes and Genomes (KEGG). The STRING tool was used to construct a Protein-Protein Interaction (PPI) network.

### Chromatin immunoprecipitation sequencing (ChIP-seq)

ChIP-seq assay was conducted as described^36^. MLO-Y4 cells were subsequently treated with SRI for 48 h, crosslinked with formaldehyde for 10 min, and treated with MNase (Micrococcal nuclease) for chromatin fragmentation for 20 min. The treated cells were ultrasonically homogenized according to the instructions of the ChIP assay kit (Beyotime, Shanghai, China). The homogenized samples were incubated with (1:100, CST#38454, Cell Signaling Technology, MA, USA) or IgG (1:50, CST#2729, Cell Signaling Technology, MA, USA) antibodies at 4°C overnight, and then with protein A/G magnetic beads at 4°C for 2 h. After eluted out from the antibody-protein A/G beads, the DNA fragments were sent for DNA sequencing immediately (Sangon Biotech Inc., Shanghai, China). The results were visualized using IGV software for peak sequences, and subsequent ChIP-qPCR assay was performed to quantify the immunoprecipitated DNA. The data were normalized to the input. ChIP-qPCR primers sequence was listed in Supplementary information, Table S3.

### Luciferase assay

Luciferase assay was performed as previously reported^37^. First, RANKL gene chromosome region containing Smad4 binding site was inserted into the pGL3 empty luciferase reporter by Liuhe BGI (Beijing, China), The pGL3_RANKL-S4BS_Luc reporter construct (2 μg/24-well) was transiently transfected into HEK293T cells using ZETA Life transfection Reagent. The pRL-null vector (0.2 μg/24-well) carrying the Renilla luciferase was co-transfected as an internal control. The transfected cells were treated with 5 μM SRI for 24 h, and luciferase activities were measured using the Dual-Luciferase® Reporter Assay (Promega, Madison, USA).

### Osteoclast counting on cancellous bones

According to the method previously reported^20^, bones were dissected from the 2-month-old TGFβRII^ΔOt^ mice and their cohort controls, fixed in 10% PBS-buffered formalin, and embedded in paraffin. Thin (5 μm) longitudinal sections at the medial distal femurs were cut by using a microtome Leica RM2255, and stained for TRAPase with a counterstain of fast green. Osteoclast counting was performed by using OsteoMetrics. The terminology and units used are those recommended by the Histomorphometry Nomenclature Committee of the American Society for Bone and Mineral Research^38^.

### Immunofluorescence

Immunofluorescence was applied to detect nuclear entry of β-catenin as previously described^39^. Briefly, WTO were cultured in 24-well plate and treated with DMSO or C91 for 48 h. After fixed in 4% paraformaldehyde for 10 min, the cells were permeabilized with 0.25% Triton X-100 in PBS-T and blocked with 5% BSA, followed by incubation with rabbit polyclonal anti-mouse β-catenin antibody (1:100, WL0926a, Wanleibio, Shenyang, China), and FITC-labeled goat anti-rabbit IgG (H + L) antibody (1:300, A0562, Beyotime, Shanghai, China). The cells were stained with DAPI, then acquired images using a fluorescence microscope (Leica, Wetzlar, Germany) with type of the objective lenses: FLUOTAR, magnification: 20X, and numerical aperture: 0.55.

### Immunohistochemistry (IHC)

The expression of the indicated proteins was visualized in paraffin section as described^20^. Briefly, sections were deparaffinized, treated with 3% H_2_O_2_ to inhibit endogenous peroxidase activity, blocked with 10% goat serum, and then incubated with polyclonal antibody; then incubated with HRP-labeled secondary anti-rabbit IgG (H+L). Color was developed with a diaminobenzidine substrate chromogen system (ZSGB-BIO). Positive cells are stained in brown, and then counterstained with hematoxylin (Biosharp, Anhui, China). Corresponding non-immune IgGs were used as negative controls.

### Statistical analysis

Data were analyzed by GraphPad Prism 8.3. Data were presented as means ± standard deviation (SD). The difference between multiple groups and two independent variables was analyzed by using One-Way ANOVA and Two-Way ANOVA, respectively. Student's *t*-test was performed between two comparable groups. Nonparametric Kruskal-Wallis test was used to compare medians instead when data distributions are not normal. A *P* value < 0.05 was considered significance for all statistical tests. Each experiment was repeated at least three times independently.

## Results

### Primary osteocytes with dominantly active β-catenin (daCO) promote osteoblast differentiation

**Figure 1:**
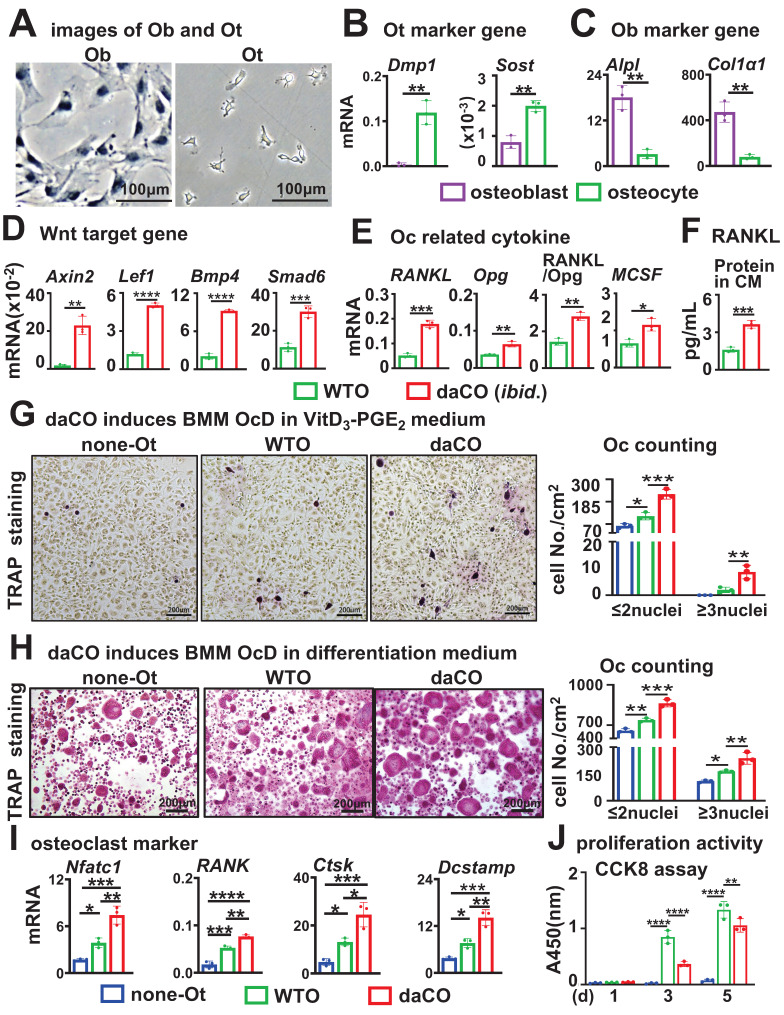
** Isolated primary osteocytes with dominantly active β-catenin (daCO) express higher levels of RANKL and Opg with increased RANKL/Opg ratio for osteoclast differentiation. A-C** The images (**A**) and marker gene expression of osteoblasts (**B**) and osteocytes (**c**). Cells were isolated and cultured from the bone marrow of the tibiae and femurs of C57BL/6 mice. **D-E** Expressions of canonical Wnt target genes (**D**) and pre-osteoclastogenic cytokines *RANKL*, *Opg*, and *MSCF* (**E**). **F** RANKL protein amount in the conditioned medium of primary osteocytes as detected by ELISA. **G-H** TRAP staining and TRAP^+^ cell counting in the co-culture of bone marrow monocytes and macrophages (BMM) and osteocytes in osteoclast supplementary medium containing VitD_3_ and PGE_2_ (**G**) or OcD medium containing 50 ng/mL RANKL and 30 ng/mL MCSF (**H**). **I** Assay for osteoclast marker gene expression. **J** Cell proliferation activity. Data were expressed as mean ± SD. Statistics: *t*-test for panels **B-F,** *p < 0.05, n=3**;** One-Way ANOVA for panels** G-I.** *p < 0.05 v.s. none-Ot, ^#^*p* < 0.05 v.s. WTO, n=3; Two-Way ANOVA for panel **J**. *p < 0.05 v.s. none-Ot, ^#^*p* < 0.05. v.s. WTO, n=3. Ob, osteoblast; Ot, osteocyte; none-Ot, no osteocytes; WTO, wild-type osteocytes; daCO, osteocytes with dominantly active β-catenin.

To examine the effect of daCO on OcD, we isolated and cultured primary osteocytes from mouse tibiae and femurs. The osteocytes were triangle in shape and 10~20 μm in size, far smaller than polygonal osteoblasts with a size of 20~80 μm (Figure 1A). As expected, compared with osteoblasts, osteocytes expressed osteocytic marker genes at much higher levels than osteoblasts (Figure 1B), and in contrast expressed osteoblastic marker genes at much lower levels (Figure 1C).

daCO had enhanced Wnt signaling with higher expression of Wnt target genes compared to wild type osteocytes (WTO) (Figure 1D). As RANKL and MCSF are pro-osteoclastogenic cytokines, essential for OcD. qPCR results showed that daCO highly expressed *RANKL* and *MCSF*, 3.5 and 1.8 times higher than WTO, respectively. The RANKL/Opg ratio in daCO was still 2.0 times higher than that in WTO (Figure 1E). Moreover, the culture medium of daCO contained RANKL and Opg proteins at 3.6 pg/ml and 24.7 pg/ml by ELISA, respectively, much higher than the two control groups resulting in 1.6-fold increase in RANKL/Opg ratio as well (Figure 1F; and Figure S**1**). Thus, daCO shows higher potential to promote OcD.

To determine whether daCO is sufficient to induce OcD, we co-cultured daCO with mice bone marrow monocytes and macrophages (BMM) in growth medium and observed the formation of multinucleated giant cells, namely osteoclasts identified by TRAP staining. After 9 days of cultivation and continuous observation, TRAP staining results showed that no osteoclasts with a nuclear number ≥ 3 were found in the co-culture; However, it is worth noting that daCO induced differentiation of osteoclast precursors (TRAP positive cells with nuclear ≤ 2), with a cell count of 89.3/cm^2^, which was 6.4 and 3.3 times higher than the control groups none-Ot and WTO, respectively (Figure S2A). This indicates that daCO by itself is not sufficient to induce OcD *ex vivo*, which may be due to the low concentration of RANKL produced in the culture medium of daCO, which is also mentioned in the co-culture of WTO and BMM^7^.

As osteoblasts increase the expression of RANKL and MCSF when treated with VitD_3_, we then added VitD_3_ and PGE_2_ to the growth medium, as applied by Nakashima et al^7^. The added VitD_3_ and PGE_2_ indeed help induce osteoclast formation in the co-culture of BMM with daCO. The TRAP staining results showed that the number of osteoclasts with a nucleus ≥ 3 in the daCO group was 8.7/cm^2^, which was 4.3 times higher than the WTO control (Figure 1G). In contrast, the supplementary VitD_3_ and PGE_2_ by themselves cannot help induce osteoclast formation in BMM without osteocytes. It is worth noting that in all three groups, supplementary VitD_3_ and PGE_2_ also help induce differentiation of osteoclast precursors with a nucleus ≤ 2. The number of TRAP positive cells was 233.3/cm^2^ in the daCO group, which was 1.7 times and 2.6 times higher than the two controls.

To effectively evaluate the effects of daCO on OcD, breakthrough of the OcD threshold, RANKL and MCSF recombinant proteins were added to the growth medium. daCO significantly increased the number of induced osteoclasts, reaching 237.7/cm^2^, which was 3.0 and 1.5 times higher than the none-Ot and WTO controls, respectively (Figure 1H); The number of induced osteoclast precursors also significantly increased, reaching 924.3/cm^2^, which was 1.8 and 1.4 times higher than the two control groups. qPCR confirmed the highest expression of osteoclast marker genes *NFATc1*, *Rank*, *Ctsk*, and *Dcstamp* in the daCO group, much higher than the two control groups (Figure 1I). These results indicate that daCO greatly promotes OcD. Hereafter, RANKL and MCSF recombinant proteins were used in the co-culture medium for the study.

In the non-contact transwell co-culture system established using transwell chambers (0.4 μm), the pro-osteoclastogenic capacity of daCO osteocytes (cultured in the upper chamber) to induce multinucleated osteoclast differentiation in BMM (cultured in the lower chamber) was 1.97- and 1.3-fold higher than in none-Ot and WTO controls, respectively (Figure S2B). This enhancement was less pronounced than that observed in direct contact co-culture, indicating that daCO-mediated osteoclast differentiation is predominantly mediated by membrane-bound RANKL and requires direct cellular contact.

CCK-8 assay of proliferation activity was performed in the co-culture of osteocytes with BMM, and both daCO and WTO linearly proliferated BMM, although daCO reduced the proliferation activity of BMM compared to WTO after 3 and 5 days of cultivation (Figure 1J).

### daCO-induced osteoclasts have high bone resorption activity

**Figure 2:**
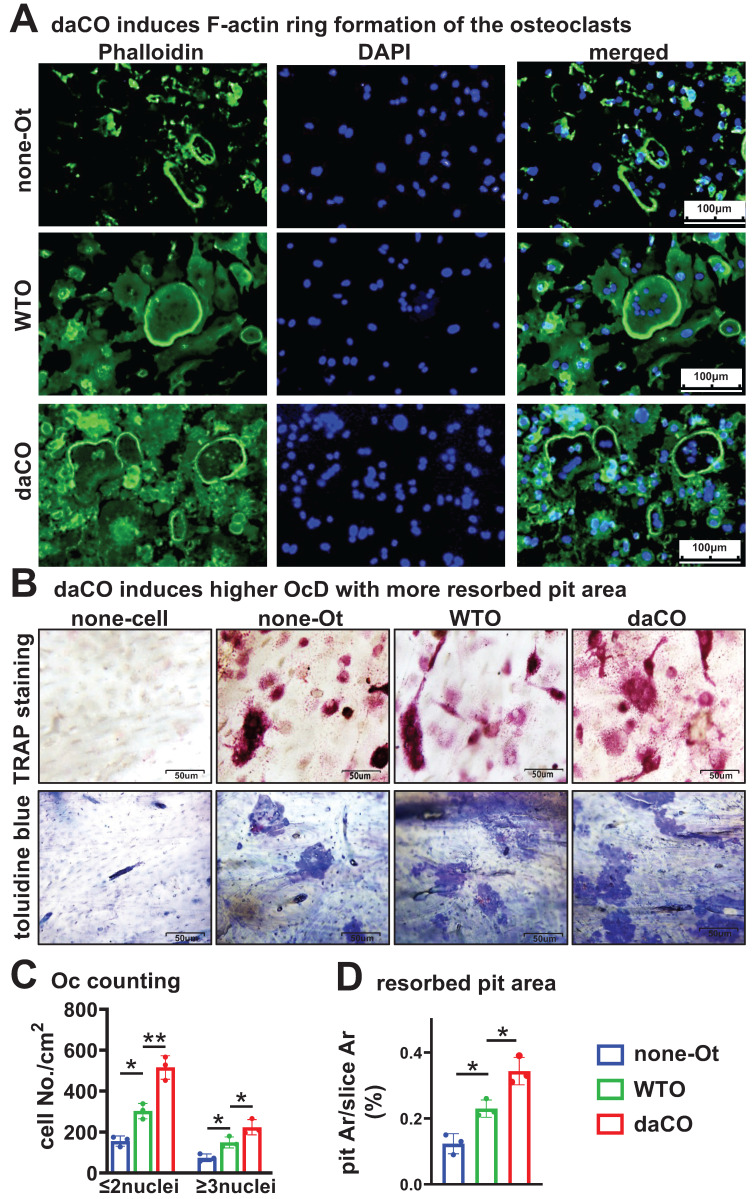
** Osteocytic Wnt-induced osteoclasts have higher bone resorptive activity**. **A-B** Phalloidin, TRAPase, and toluidine blue staining of bone slices with co-cultured osteocytes and BMM. **C** TRAP positive cell counting. **D** Resorbed pit area calculation on toluidine blue stained bone slices by OsteoMetrics. none-Ot, no osteocytes; WTO, wild-type osteocytes; daCO, osteocytes with dominant active β-catenin. Data were expressed as mean ± SD, *p < 0.05 v.s. none-Ot, ^#^*p* < 0.05. v.s. WTO by One-Way ANOVA, n=3.

To evaluate the bone resorption activity of daCO-induced osteoclasts, we used a traditional pit assay^33^ to measure the formation of the F-actin ring, which is the characteristic of mature osteoclasts with bone resorption ability^40^, ^41^. On the bone slices, YF 488-Phalloidin staining showed daCO induced more osteoclasts with F-actin rings than that of the two controls under fluorescent microscope (Figure 2A). TRAP staining showed the largest number of daCO-induced osteoclasts at 223.8/cm^2^, which was 1.5 and 3.0 times that of the two control groups. So does the number of TRAP^+^ osteoclast precursors, reaching 515.4/cm^2^ (Figure 2, B and C). After the cells were erased from the bone slices, Toluidine blue staining visualized resorbed pit area. As measured by OsteoMetrics, the biggest pit area was in the daCO group, with a resorbed pit Ar./slice Ar. 12.3%, which was 2.8 and 1.5 times higher than those of the none-Ot and WTO groups, respectively (Figure 2D), indicating that the induced osteoclasts by daCO have the strongest bone resorption activity.

### Osteocytic Wnt generates RANKL for osteoclast differentiation

**Figure 3:**
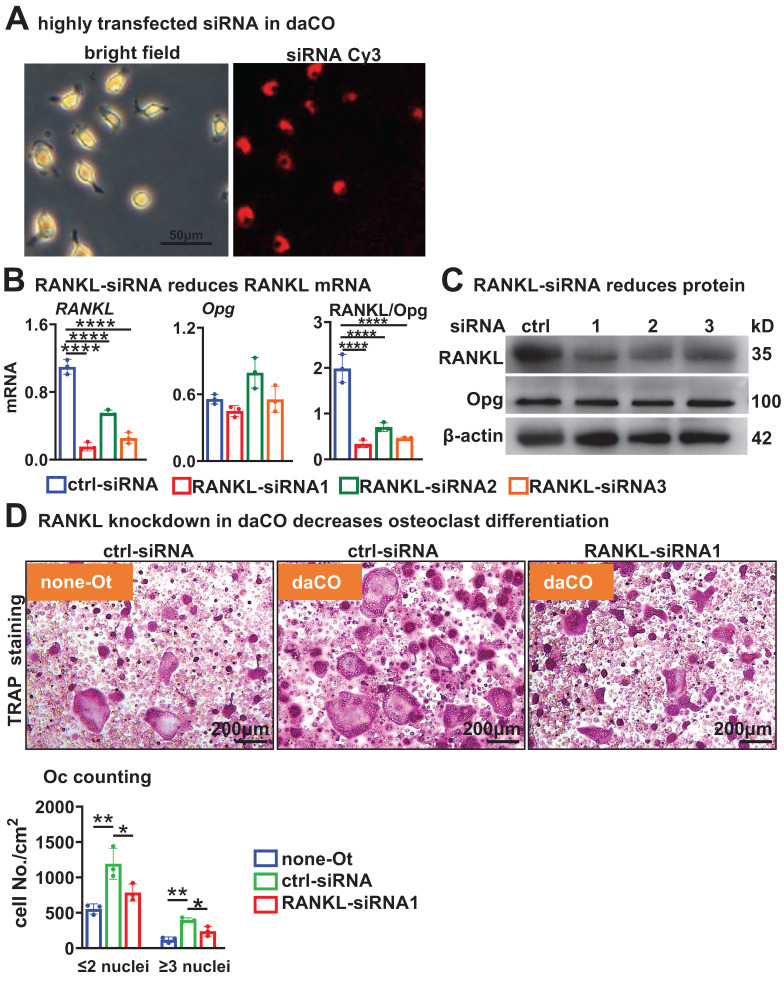
**RANKL knockdown reduces daCO-induced OcD. A** Transfection efficiency of Cy3-labeled control siRNA in daCO. Images were taken under microscope with bright field and red fluorescence. **B-C** qPCR and Western blotting for the expression of RANKL and Opg at mRNA (**b**) and protein (**C**) levels in daCO after transfected with siRNAs for 48 h. **D-E** TRAP staining (**D**) and TRAP positive cell counting (**E**) in the co-culture of BMM and osteocytes. none-Ot, no osteocytes; WTO, wild-type osteocytes; daCO, osteocytes with dominantly active β-catenin. Data were expressed as mean ± SD. *p < 0.05 v.s. none-Ot, ^#^*p* < 0.05. v.s. ctrl-siRNA by One-Way ANOVA, n=3.

To test how daCO promotes OcD, we knocked down *RANKL* expression in osteocytes by using RANKL interfering RNA (RANKL-siRNA). Under the fluorescence microscope, control-siRNA labelled with cy3 was transfected into primary daCO at high efficiency of ≥90% by using ZETA Life transfection reagent (Figure 3A). qPCR analysis showed that each of the 3 RANKL-siRNAs downregulated *RANKL* expression by 85.9%, 49.6%, and 76.7%, respectively; however, they did not affect the expression of *Opg*, resulting in decreased RANKL/Opg ratio by 64.4% to 82.9% (Figure 3B). Moreover, Western blotting results showed a similar decrease of RANKL protein (Figure 3C). RANKL knockdown markedly reduced the OcD enhanced by daCO (Figure 3, D and E). Osteoclasts were formed at 237.2/cm^2^ in siRNA1-treated daCO group, which was decreased by 40.1% compared to the control-siRNA group. Similarly, the number of TRAP^+^ osteoclast precursors were 786.5/cm^2^ in siRNA1-treated daCO group, which was decreased by 34.0% compared to the control-siRNA group. These results suggest that daCO promotes OcD by producing RANKL.

### Osteoclast differentiation is the most associated KEGG pathway in daCO with increased TGFβ signaling

**Figure 4:**
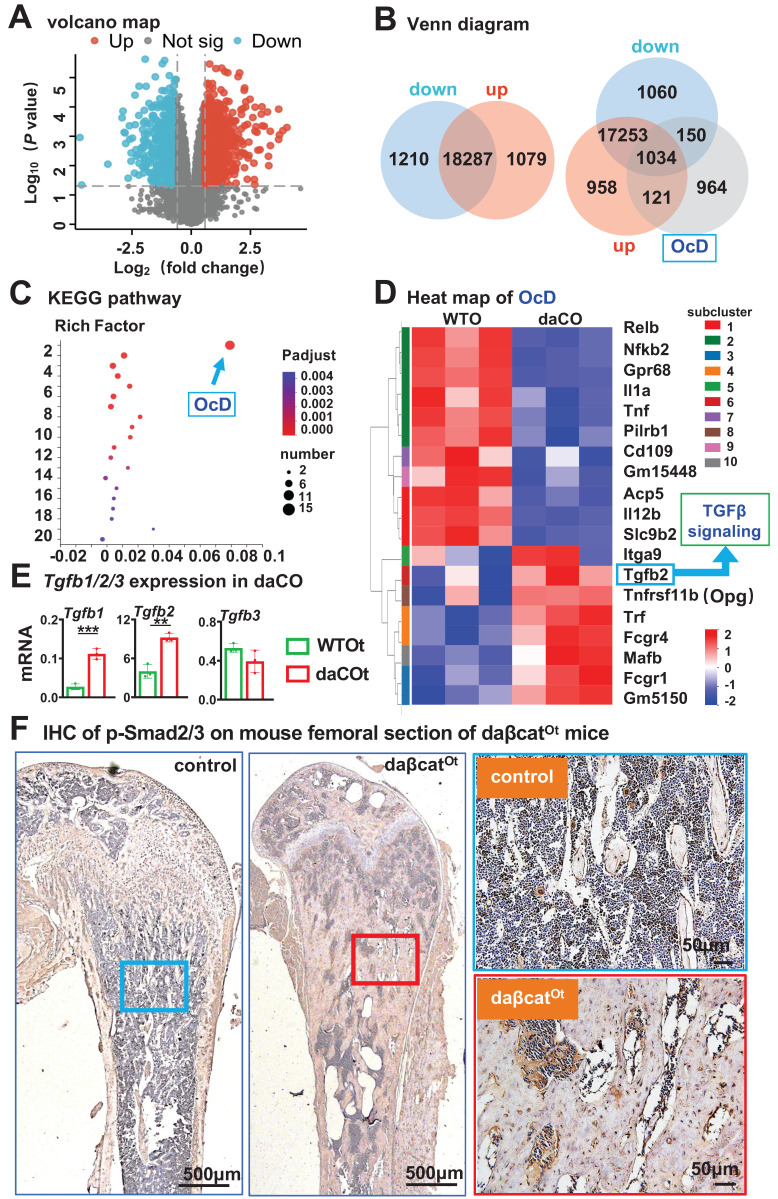
** RNA-seq of daCO. A** Volcano plot of differentially expressed genes between daCO and WTO. **B** Identify differential genes between the daCO and WTO groups and genes associated with OcD were identified by Venn diagram analysis. **C** KEGG pathways enriched from differentially expressed genes of daCO versus WTO. Ordinate: 1. OcD, 2. Leishmaniasis, 3. Tuberculosis, 4. Cytokine-cytokine receptor interaction, 5. Inflammatory bowel disease, 6. MAPK signaling pathway, 7. Rheumatoid arthritis, 8. Pertussis, 9. AGE-RAGE signaling pathway, 10. C-type lectin receptor signaling pathway, 11. Type I diabetes mellitus, 12. Hematopoietic cell lineage, 13. Toll-like receptor signaling pathway, 14. Influenza A, 15. Hypertrophic cardiomyopathy, 16. Toxoplasmosis, 17. Le-gionellosis, 18. Graft-versus-host disease, 19. Prion diseases, 20. Human T-cell leukemia virus 1 infection. **D** Heatmap of OcD-related genes. **E** Validating the expression of TGFβ ligands *Tgfb1*, *Tgfb2*, and *Tgfb3* in daCO. *p < 0.05 by *t*-test, n=3. **F** Immunohistochemical assay of p-Smad2/3 on the longitudinal cross section of mouse femurs. Square boxes are the enlarged area in the diaphysis. * *p* < 0.05 by *t*-test, n=3.

To explore the molecular mechanism by which daCO produces RANKL, we investigated daCO transcriptome by RNA-seq. Box plots showed that the information of each sample is on the same baseline post-normalization, indicating suitable comparability among these data (Figure S3A). Principal component analysis (PCA) reveals a significant difference and separation between daCO and WTO samples (Figure S3B), indicating a reliable relation. As compared to WTO, differentially expressed genes of daCO were identified as a total of 2,289 genes, of which 1,079 genes were up-regulated and 1,210 genes were downregulated (Figure 4, A and B). By intersecting OcD-related genes retrieved from Genecards with the differentially expressed genes, we identified 121 upregulated and 150 downregulated differentially expressed genes (Figure 4B). KEGG pathway enrichment highlighted OcD with the most enriched differentially expressed genes including TGFβ, TNFα, IL1, Opg, and Ig-like R/FcRy in the upstream of OcD, and NFκB, CTSK, TRAP, and calcitonin receptor in the downstream of OcD (Figure 4C; and Figure S4). Heatmap analysis of osteoclast-related genes revealed higher expression of *Tgfb2* (Figure 4D; and Figure S3C), and qPCR analysis of daCO verified 4.1 and 2.3-fold increase in the expression of *Tgfb1* and *Tgfb2*, respectively compared to WTO, whereas *Tgfb3* expression was comparable in daCO versus in WTO (Figure 4E). Protein-Protein Interaction analysis (PPI) also shows that Tgfb2 is a critical regulatory node among the differentially expressed protein related to OcD (Figure S5).

Furthermore, the expression level of p-Smad2/3, an active transcription factor that is a marker of the TGFβ signaling pathway, was increased in the femoral osteocytes from daβcat^Ot^ mice compared to their littermate control mice (Figure 4F). In contrast, no increase in p-Smad2/3 expression levels were detected in femoral osteoblasts of i-daβcat^Ob^ mice induced to express the same daβ-catenin in osteoblasts by Col1-2.3kb-CreERT2 compared to their littermate control mice (Figure S6A), nor was there an increase in the expression of the *Tgfb1* and *Tgfb2* genes in the femurs and tibiae of the i-daβcat^Ob^ mice compared to their littermate control mice (Figure S6B). Moreover, Wnt agonist C91-treated calvaria cells of wildtype mice did not exhibit increased expression of the *Tgfb1* and *Tgfb2* genes as well compared to DMSO-treated calvaria cells (Figure S7). These data suggest that TGFβ signaling is specifically regulated by osteocytic Wnt rather than osteoblastic Wnt.

### Wnt agonist recapitulates its role on TGFβ signaling and RANKL expression in wild-type osteocytes

**Figure 5:**
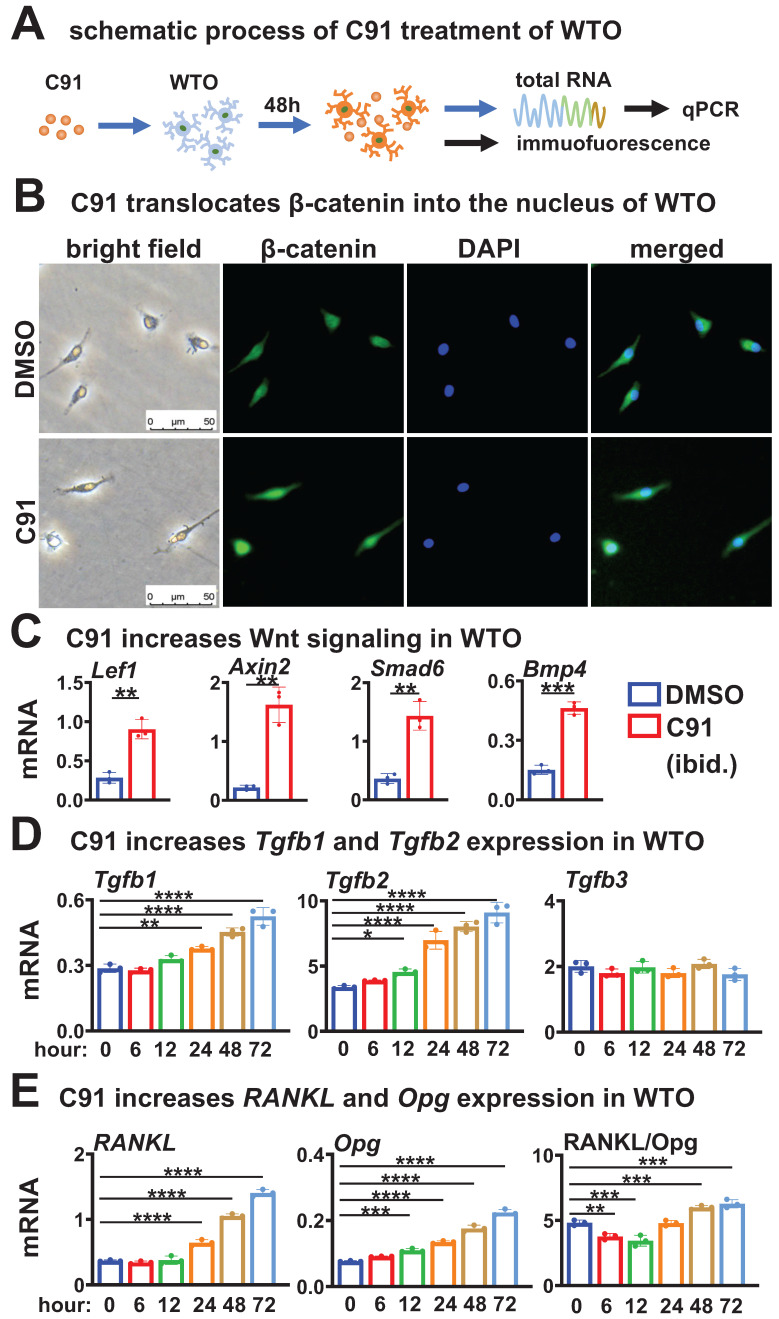
** Wnt agonist C91 time-dependently increases the expression of *Tgfb1*, *Tgfb2*, and *RANKL* in WTO. A** Schematic treatment of C91 in WTO. **B** Immunofluorescence detects β-catenin in C91-treated WTO for 48 h. **C** Wnt target gene expression in C91-treated WTO**. D-E** Expression of *Tgfb1*, *Tgfb2*, and *Tgfb3* (**D**) and *RANKL*, *Opg*, and RANKL/ Opg ratio (**E**) in C91-treated WTO for indicated time. Data were expressed as mean ± SD. *p < 0.05 v.s. DMSO by One-Way ANOVA., n=3. WTO, wild-type osteocytes; daCO, osteocytes with dominantly active β-catenin.

We next investigated whether the upregulation of *Tgfb1/2* and *RANKL* expression by daCO could be replicated in WTO under stimulation of Wnt agonists, particularly the relationship between the TGFβ signaling pathway and RANKL generation. After comparative evaluation, we prioritized an alternative Wnt agonist C91 (Figure 5A) over recombinant Wnt3a due to its demonstrably enhanced stability and significantly stronger promotion of osteoclast differentiation (Figure S8), ensuring consistent responses in downstream mechanistic studies. C91 increased β-catenin expression and nuclear entry in WTO by immunofluorescence assay after 48 h of treatment at a dose of 5 μM compared with DMSO control (Figure 5B). Compared to DMSO treatment, C91 treatment enhanced canonical Wnt signaling with higher expression of Wnt target genes (Figure 5C). Strikingly, C91-activated Wnt signaling elevated the expression of* Tgfb1* and *Tgfb2* in a time-dependent manner, but had no effects on the expression of* Tgfb3* (Figure 5D). Meanwhile, *RANKL* expression was also increased in a time-dependent manner, with an increase in the RANKL/Opg ratio after 48 h of C91 treatment (Figure 5E). These results suggest that osteocytic Wnt upregulates the expression of *Tgfb1* and *Tgfb2* in a time-dependent manner to activate TGFβ signaling, which may be associated with the increase in RANKL expression and RANKL/Opg ratio.

Following establishment of an *in vivo* C91 administration model via paravertebral multipoint injection (60 mg/kg/day × 14 days), bone histomorphometric analysis revealed significant increases in osteoclast parameters within femoral trabecular bone: osteoclast number per bone perimeter (N.Oc/B.Pm) and osteoclast surface per bone surface (Oc.S/BS) were elevated by 60% and 44%, respectively, compared to controls (data not shown). Immunohistochemical analysis confirmed substantial upregulation of RANKL protein expression in osteocytes (Figure S9A), with concurrent specific activation of p-Smad2/3 signaling nodes in trabecular osteocytes (Figure S9B). These collective findings provide *in vivo* validation that the TGFβ/Smad pathway functions as the central molecular switch mediating activation of the Wnt-to-RANKL signaling axis.

### Osteocytic TGFβ specifically upregulates RANKL expression for osteoclast differentiation

**Figure 6:**
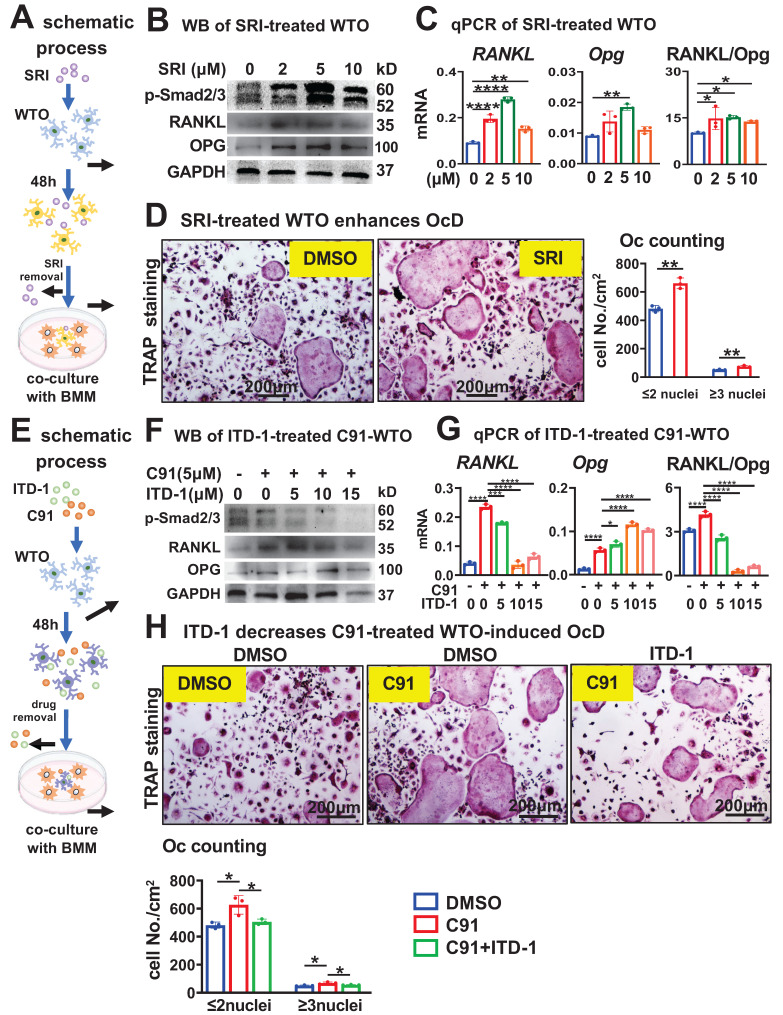
** TGFβ signaling specifically increases RANKL expression in time- and dose-dependent ways in WTO for OcD. A-D** Activation of osteocytic TGFβ signaling enhances RANKL expression and OcD. **A** Pattern diagram: TGFβ signaling agonist SRI treatment of WTO on OcD. **B-C** SRI treatment for the regulation of RANKL, Opg, and RANKL/Opg expression at protein (**B**) and mRNA (**c**) levels in WTO. **D** TRAP staining and TRAP^+^ cell counting in the co-culture of BMM and SRI-treated WTO. **E-H** inactivation of osteocytic TGFβ signaling reverses SRI-enhanced RANKL expression and OcD. **E** Pattern diagram: TGFβ signaling antagonist ITD-1 treatment of C91-WTO on OcD. **F-G** ITD-1 treatment for the regulation of RANKL, Opg, and RANKL/Opg expression at protein (**F**) and mRNA (**G**) levels in C91-treated WTO. **H** TRAP staining and TRAP^+^ cell counting in the co-culture of BMM and ITD-1-treated C91-WTO. Data were expressed as mean ± SD. WTO, wild-type osteocyte; SRI, TGFβ signaling agonist; ITD-1, TGFβ signaling antagonist. *p<0.05 v.s. DMSO, ^#^*p*<0.05 v.s. C91 by One-Way ANOVA, n=3.

We next investigated whether osteocytic TGFβ signaling specifically increases RANKL expression (Figure 6A). Compared to DMSO control, treatment of WTO with the TGFβ signaling activator SRI for 48 h dose-dependently increased the expression level of p-Smad2/3 (Figure 6B), which is the marker of activation of TGFβ signaling. SRI dose-dependently increased *RANKL* expression in WTO as well, and although there was a slight decrease at 10 μM compared to 5 μM. As SRI leading to a similar increase in the RANKL/Opg ratio between 2~10 μM SRI treatments (Figure 6C). These were also confirmed by Western blotting for RANKL protein in a similar way (Figure 6B). Moreover, compared with DMSO control, WTO treated with SRI for 48 h significantly promoted OcD of BMM when co-cultured with BMM. TRAP staining showed that the SRI-treated group had a 1.4-fold increase in the number of osteoclasts and osteoclast precursors by compared to DMSO control (Figure 6D).

To further validate TGFβ-mediated regulation across different agonist modalities, we additionally treated WTO osteocytes with recombinant Tgfb1 (0-5 ng/mL)^42^ and Tgfb2 (0-15 ng/mL)^43^ for 48 hours followed by agonist withdrawal. In co-culture with BMM, both low-dose TGFβ ligands significantly enhanced osteoclast differentiation, with TRAP quantification identifying 1 ng/mL as the optimal concentration for both Tgfb1 and Tgfb2 (Figure S10A). Western blot analysis at these optimal concentrations confirmed potent activation of TGFβ signaling, as evidenced by upregulated p-Smad2/3 expression following treatment with 1 ng/mL Tgfb1 and 1 ng/mL Tgfb2 (Figure S10B). Critically, qPCR confirmed significant RANKL transcriptional induction (Figure S10C). This complementary data reinforces that TGFβ pathway activation - whether via small molecule agonists or native ligands - consistently upregulates osteocytic RANKL production to promote osteoclastogenesis.

On the other hand, we tested whether inhibition of TGFβ signaling in osteocytes affects osteocytic Wnt-enhanced OcD. In this case, WTO was treated with both the TGFβ RII inhibitor ITD-1^44^ and the Wnt agonist C91 for 48 h as scheduled (Figure 6E). Western blotting showed that C91 increased p-Smad2/3 protein levels in the WTO group, however ITD-1 decreased C91-increased p-Smad2/3 protein levels in a dose dependent manner (Figure 6F). Moreover, ITD-1 also decreased *RANKL* expression and RANKL/Opg ratio in a dose dependent manner as well, which were increased by C91 (Figure 6, F and G), suggesting that the specific inhibition of C91-activated TGFβ signaling by ITD-1 reduces RANKL expression.

Furthermore, 10 μM ITD-1-treated WTO were co-cultured with BMM for OcD test. TRAP^+^ cell counts showed that C91-treated WTO produced 68.8/cm^2^ osteoclasts and 627.0/cm^2^ osteoclast progenitors, respectively, which were 1.4-fold and 1.3-fold than those of the DMSO-treated osteocyte controls. Importantly, inhibition of TGFβ signaling by ITD-1 reduced the number of osteoclasts and their progenitors, respectively (Figure 6H), suggesting that osteocytic Wnt-induced OcD requires TGFβ signaling.

### TGFβ signaling activates RANKL transcription via physical binding of Smad4 to the RANKL promoter region

**Figure 7:**
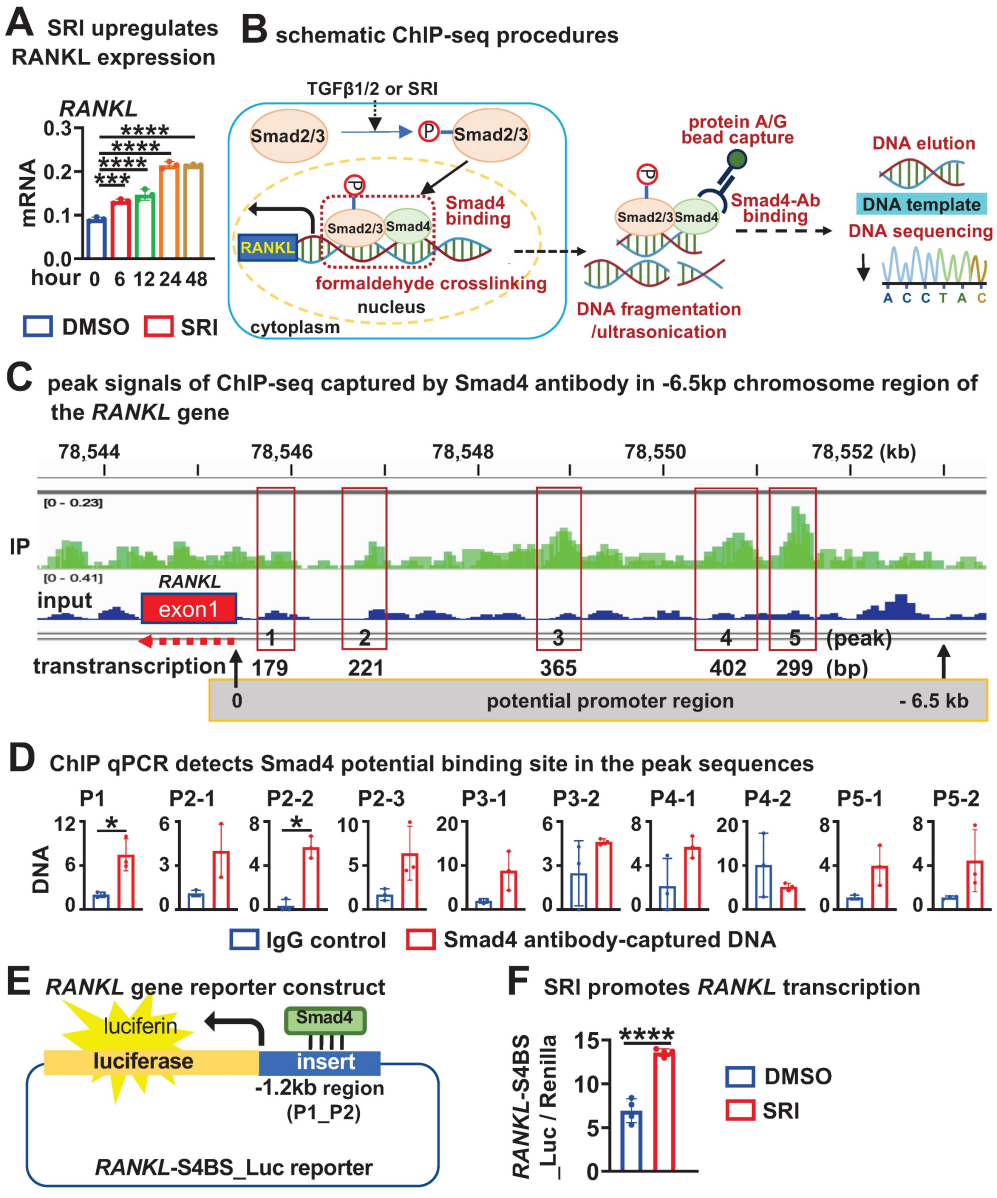
** TGFβ signaling promotes RANKL transcription through physical interaction of Smad4 in the promoter region. A** SRI time-dependently increases *RANKL* expression in WTO. **B** Schematic ChIP-seq procedures with Smad4 antibody. **C** IGV visualization of ChIP-seq results for peak signals screened at -6.5 kb chromosome region of the *RANKL* gene. **D** ChIP-qPCR with the primers designed within the 5 peak sequences for Smad4 binding sites. **E** RANKL gene reporter construction of the -1.2 kb chromosome region containing peaks 1 and 2 with Smad4 binding site. **F** SRI stimulates physical binding of Smad4 to the P1_P2 site of the RANKL-S4BS_Luc reporter. Data were expressed as mean ± SD for panels **A**, **D**, and **F**. WTO, wild-type osteocyte. *p < 0.05 by One-Way ANOVA, n=3 for panel **A**. *p < 0.05 by *t*-test n=3 for panels **D** and **F**.

We found that SRI increases RANKL expression in a time-dependent manner, with this increase occurring as early as 6 h after SRI treatment (Figure 7A). Next, we investigated whether TGFβ signaling could directly activate *RANKL* transcription by ChIP-seq (Figure 7B). We obtained DNA bound to Smad4 (a core mediator of TGFβ/BMP signaling pathways) from SRI-treated osteocytic cell line MLO-Y4 cells for 48 h and performed ChIP-sequencing analysis. Based on IGV analysis of signals with binding potential, we selected 5 peak sequences that increased relative to the Input set (Figure 7C). Subsequent ChIP-qPCR detected significantly amplified peak 1 (P1) and peak 2 (P2-2) sequences (Figure 7D).

We then inserted the P1 and P2 sequence regions within the -1.2kb region of RANKL gene into a pGL3-luciferase empty report construct to generate pGL3_RANKL-P1-P2_luc reporter construct. After transfection of the report construct into HEK293T cells, their transcription activity was detected after 48 h of SRI treatment (Figure 7E). SRI treatment increased the transcription of pGL3_RANKL-P1-P2_luc by 2.1-fold compared with DMSO treatment (Figure 7F), suggesting that TGFβ signaling directly activates RANKL transcription by transcription factor Smad4 binding to the P1-P2 locus in the -1.2kb region of the *RANKL* gene.

### Mice with disrupted TGFβ-signaling in osteocytes recapitulate reduced RANKL expression and defective osteoclastogenesis in cancellous bone

**Figure 8:**
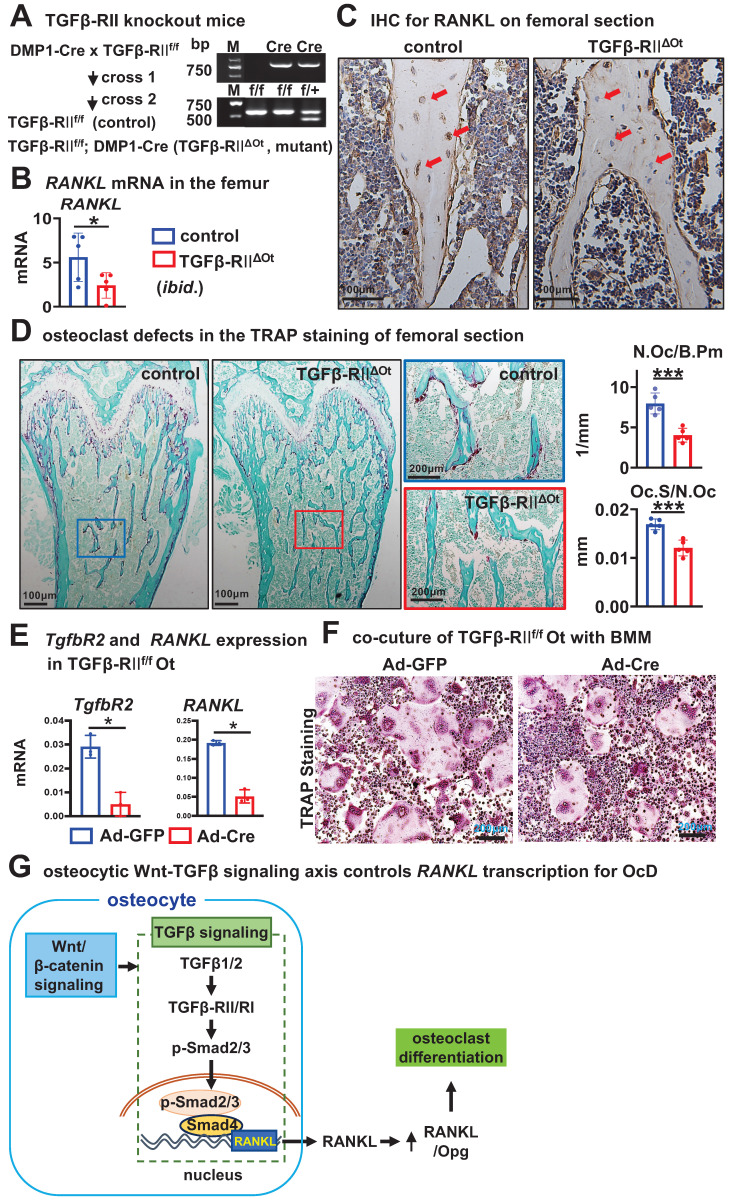
** Osteocytic deletion of TGFβ signaling decreases RANKL expression and osteoclast number and activity in mouse cancellous bone**. **A** Generation of mutant mice with osteocytic deletion of TGFβ signaling by 2 crosses of the TGFβRII floxed mice with DMP1-8kb-cre mice. Right, genotyping of the mutant mice. **B-C** Expression of RANKL at mRNA (**B**) and protein (**C**) levels in the femurs. **D** TRAP staining and measurement of osteoclast number and activity in the longitudinal femoral sections. Square boxes are the enlarged area in the diaphysis. N.Oc/B.Pm and Oc.Pm/N.Oc are the number of osteoclasts per bone perimeter and the eroded surface of bone per osteoclast, respectively. **E** Validating the expression of *Tgfbr2* and *RANKL* in Ad-Cre/GFP-treated TGFβ-RII^f/f^ Ot. **F** TRAP staining of the co-culture of BMM and Ad-Cre/GFP-treated TGFβ-RII^f/f^ Ot. **G** A model for the osteocytic Wnt-TGFβ signaling axis controls RANKL transcription for OcD. Data were expressed as mean ± SD, *p < 0.05 v.s. control mice by *t*-test, n=5.

To validate the *in vivo* function of osteocytic TGFβ signaling for OcD, we crossed DMP-8kb-Cre mice twice with the only TGFβ receptor II exon 4-floxed mice to generate mutant mice (TGFβ-RII^f/f^; DMP-8kb-Cre, i.e., TGFβ-RII^ΔOt^) and their littermate controls (TGFβ-RII^f/f^), and the only osteocyte-specific deletion of TGFβ signaling was confirmed by genotyping (Figure 8A). Compared with the control mice, TGFβ-RII^ΔOt^ mice had lower mRNA and protein levels of RANKL in femoral cancellous osteocytes as determined by qPCR and immunohistochemistry (Figure 8, B and C). TRAP staining of longitudinal sections of femurs from mutant mice showed a reduction in the number of osteoclasts per bone perimeter (N.Oc /B.Pm) at 3.0/mm and a shortening of the length of individual osteoclasts (Oc.S/N.Oc) at 0.016 mm, by 48.2% and 33.3%, respectively, as compared with controls (Figure 8D). Additionally, osteocytes isolated from TGFβRII^f/f^ mice and cultured *in vitro* were subjected to TGFβ type II receptor knockout via Ad-Cre adenoviral infection. qPCR analysis confirmed that Ad-Cre-infected osteocytes exhibited significantly reduced *Tgfbr2* gene expression accompanied by decreased RANKL transcription (Figure 8E). When these receptor-deficient osteocytes were co-cultured with wild-type BMM, they demonstrated substantially impaired osteoclastogenic capacity compared to Ad-GFP controls, as evidenced by reduced TRAP^+^ multinucleated cell formation (Figure 8F). These data demonstrated that deletion of TGFβ signaling in osteocytes resulted in defects in OcD and bone resorption, validating the function of osteocytic TGFβ signaling in the control of RANKL expression and OcD.

## Discussion

Wnt mice with daCO unexpectedly demonstrated higher expression of RANKL and higher OcD and bone resorption^20^. To clarify this function and its underlying cellular and molecular mechanisms, we isolated and cultured primary daCO from the Wnt mice, and the results confirmed that daCO displays higher expression of OcD-related factors, such as RANKL, Opg, and MCSF with an increased RANKL/Opg ratio, enhancing BMM OcD and bone resorption. These results can be reproduced by using Wnt agonist C91-treated osteocytes of wild-type mice. RANKL siRNA knocks down the expression of RANKL to reduce daCO-enhanced OcD, suggesting an osteocytic Wnt-RANKL relationship for this regulation of OcD. To explore how Wnt regulates the expression of RANKL in osteocytes, RNA-seq analysis of daCO was performed and the results revealed that the enrichment of KEGG pathways and Heatmap analysis of the regulated expression of genes respectively highlight TGFβ signaling with highest potential for OcD and its upregulated ligands *Tgfb1/2* that were verified by qPCR. In fact, TGFβ signaling transduction increases RANKL expression in time- and dose-dependent manners, respectively, increasing the RANKL/Opg ratio for OcD. Conversely, inhibition of osteocytic TGFβ signaling reverses all these effects. Mechanistically, ChIP-seq assay was performed to screen the 6.5kb chromosome area before the RANKL gene, revealing a physical binding site of canonical TGFβ signaling central player the transcription factor Smad4 at the -1.2kb region of the *RANKL* gene. qPCR and dual luciferase assay with the -1.2kb region confirmed that TGFβ signaling directly controls the transcription of RANKL gene. Finally, knocking out the only type II receptor of TGFβ signaling in osteocytes *in vivo* reduces RANKL expression both at mRNA and protein levels, resulting in a marked decrease in osteoclast number and bone resorption capacity. Thus, we found that osteocytic Wnt activates TGFβ signaling to initiate the transcription of osteoclastogenic cytokine RANKL and enhance the development of BMM into osteoclasts for bone resorption.

This study demonstrates that osteocytic Wnt promotes, rather than inhibits OcD and bone resorption, which is a broad conceptional advance. Because it has been reported that osteoblastic Wnt signaling does not affect bone formation; instead, only upregulates the expression of the Wnt target gene, Opg without regulating RANKL expression, resulting in a decrease in the RANKL/Opg ratio to inhibit osteoclastogenesis and increase bone mass^12^. Therefore, osteoblastic Wnt has only this single biological function. The findings of the present study add to the knowledge of developmental biology, in which Wnt signaling has a second function in upregulating the expression of RANKL with increased RANKL/Opg ratio to enhance OcD. This suggests that bone cells have different functions at different developmental stages, reflecting the temporal and spatial nature of Wnt signaling. This is a significant discovery of the physiological function of osteocytes.

Based on the non-contact Transwell co-culture findings, the daCO phenotype in osteocytes promotes osteoclast differentiation predominantly through contact-dependent signaling mediated by membrane-bound RANKL (mRANKL), while secretory factors may contribute via auxiliary pathways. Building upon this, subsequent investigations will focus on elucidating how co-stimulatory molecules (e.g., ICAM-1/JAG1) spatially cooperate with RANKL to orchestrate dynamic assembly at the cellular contact interface. This will unravel the molecular principles governing signal amplification during osteoclast-osteocyte crosstalk, ultimately informing the development of precision-targeted interventions for osteoporosis.

Wnt mice with daCO exhibits increases in bone anabolism and bone resorption^20^. Apparently, the latter function is in contrast to mice expressing the same daβ-catenin in osteoblasts. To date, there is no direct evidence in the field that canonical Wnt signaling in bone cells promotes OcD, as this phenomenon may also arise indirectly, e.g., massive bone formation is often accompanied by systematic enhancement of bone resorption, the so-called “bone formation coupled to bone resorption”. In addition, in the past we have even suggested that a Sost/sclerostin-dependent pattern promotes RANKL expression. Therefore, only after isolating and culturing osteocytes can we logically elucidate the above functions of osteocytic Wnt and analyze the underlying mechanisms accordingly. More importantly, osteocytic Wnt has a dual function of bone formation and bone resorption, which may have translational value, as discussed below.

Secondly, we innovatively found the underlying mechanism by which osteocytic Wnt promotes OcD mainly by activating TGFβ signaling that directly activates the transcription of *RANKL* gene by physical interactions of the TGFβ signaling transcription factor Smad4 with the promoter region of the RANKL gene. However, in osteoblasts, activation of Wnt signaling did not upregulate *Tgfb1/2* expression or increase p-Smad2/3 levels, so osteoblastic Wnt does not activate TGFβ signaling, moreover, osteoblastic Wnt does not affect RANKL expression as previously reported^12^, ^17^. In addition, *RANKL* expression was upregulated in WTO as shortly as 6 h and in a time-dependent manner after the stimulation of TGFβ signaling agonist SRI, leading to an increase in OcD; on the other hand, inhibition of TGFβ signaling by ITD-1 reversed the upregulation of RANKL expression, decreasing both the RANKL/Opg ratio and OcD. Supportively, Neha *et al.* found that TGFβ signaling is an intrinsic regulator of perilacunar/canalicular remodeling (PLR) of osteocytes in mice. Deletion of TGFβ-RII, the only TGFβ type II receptor in osteocytes and osteoclasts, using DMP1-10kb-Cre impairs the PLR, thereby decreasing RANKL expression without affecting Opg expression, resulting in a lower RANKL/Opg ratio and thus a decrease in the number of osteoclasts to increase bone mass. All in all, this study confirms that the TGFβ signaling may directly stimulate the transcriptional activation and expression of RANKL to regulate OcD in the osteocyte.

Again, the mechanism of TGFβ signaling on the regulation of RANKL expression has not yet been elucidated. Moreover, under inflammatory conditions, TGFβ initiates effects followed by induction of osteoclastogenesis also in response to TNFα, independently of the physiological RANKL pathway^45^, ^46^. Lack of TGFβ signaling in macrophages inhibits inflammatory osteoclastogenesis and bone resorption *in vivo*, demonstrating a direct role of TGFβ in osteoclastogenesis^46^.

Promotion of RANKL expression by osteocytic Wnt signaling should be a sufficient but not necessary condition, as the inactivation of osteocytic Wnt signaling does not alter RANKL expression, it only reduces the expression of the target gene Opg^47^. Thus, inactivation of Wnt signaling in osteocytes also results in an increase in RANKL/Opg ratio, as well as higher OcD and bone resorption. Notably, although inactivation and activation of osteocytic Wnt signaling produce similar bone resorption-enhancing phenotypes, their molecular bases are significantly different, as evidenced by their different regulatory functions on RANKL expression, in addition to decreased or increased expression of the Wnt target gene Opg. In osteocytes, inactivation of Wnt signaling does not reduce RANKL expression, however, activation of Wnt signaling takes at least 48 h to increase RANKL expression. We speculate that the phenotype of Wnt signaling inactivation in osteocytes is similar to that of aged bone, which exhibits inhibition of Wnt signaling, increased bone resorption, and osteoporosis; however, the phenotype of Wnt signaling activation in osteocytes is more similar to that of adolescents, characterized by intense bone formation and resorption during early bone development^48^, ^49^. Therefore, we suggest that activating the Wnt signaling of osteocytes can reproduce juvenile characteristics, thus targeting osteocytes and maintaining a stable Wnt signal intensity is beneficial for elderly bone health.

This study reveals the Wnt-TGFβ-RANKL cascade as a core regulatory axis for osteoclast differentiation mediated by osteocytes. However, attention must be given to the potential retention of microenvironment-derived epigenetic memory/metabolic legacy in osteocytes isolated from high/low bone mass models, which may confound causal pathway associations. To precisely resolve bone formation-resorption coupling phenomena, future investigations will implement conditional temporally-controlled knockout strategies: Utilizing the DMP1-CreERT2 mouse model for adult-stage induction of target gene mutations to minimize developmental skeletal compensation, while integrating single-cell multi-omics to identify resistant osteocyte subpopulations and their molecular signatures. This approach will pinpoint novel therapeutic targets for memory-erasing interventions in osteoporosis.

In fact, the function of osteocytic Wnt goes far beyond promoting osteoclast differentiation. The main function of osteocytic Wnt is to promote bone formation, including osteoblast differentiation. This is also different from the function of osteoblastic Wnt, which solely enhances its target Opg expression to inhibit OcD. However, in any case, there is no evidence to suggest the osteogenic effect of osteoblastic Wnt^11^. Instead, because osteoblasts are the microenvironment of hematopoietic stem cells, maintaining their homeostasis, the Wnt signaling of osteoblasts increases the expression of Notch ligand Jag1, causing an increase in the Notch signaling in hematopoietic stem cells to change their fate by resulting in acute myeloid leukemia and death of mice 4-6 weeks after birth^50^. In contrast, the Wnt signaling in osteocytes does not produce leukemia in mice, and Wnt mice grow well with soft food feeding^20^. When we tapped into the osteogenic mechanisms by which osteocytic Wnt facilitates osteoblast differentiation, we found that decellularized matrix^30^, extracellular vesicles (in preparation for publication), and Wnt targets such as Notch ligand Dll4^51^ and BMP7^52^ etc are the osteogenic microenvironments of osteocytic Wnt. These factors can be used as bioactive materials to control cell fate, and promote bone formation, bone resorption, angiogenesis, and neurogenesis in the reconstructed 3D modules, thus to be able to form authentic bones for orthopedic purposes.

Bone formation couples with bone resorption, which may release osteogenic factors for bone formation^53^-^55^. When studying the osteogenic factors of daCO, we found that daCO decellularized matrix not only promotes osteoblast differentiation, but also promotes osteoclast formation, which may recruit stem cells for angiogenesis and neurogenesis. On this basis, we reconstructed a simulated bone with metabolic activities^30^. This is an important step in the artificial reconstruction of authentic bones, which is an effective simulation application of osteocytic Wnt with dual functions. We believe that the dual functions possess greater translational and practical value. Currently, widely prescribed bisphosphonates increase bone mass by inhibiting bone resorption. However, this approach perpetuates the retention of aged bone while suppressing new bone turnover, ultimately impairing intrinsic repair mechanisms and allowing microdamage accumulation that elevates risk for atypical femoral fractures^56^. In contrast, dual-functional agents achieve synchronized bone mass augmentation with quality optimization, thereby overcoming the fundamental limitation of conventional therapeutics - the disparity between bone mass gain and quality enhancement - through coordinated osteoanabolic-anti-resorptive synergy. Especially among clinical osteogenic drugs, such as PTH, PTHrP, and sclerostin antibodies, only PTH has the dual functions, but unfortunately, its continued use carries a risk of tumor development, as does osteoblastic Wnt^50^, while osteocytic Wnt does not^20^. After 24 h of treatment with Wnt agonists of small molecule drugs, the osteocyte becomes a safe osteogenic microenvironment with these functions of osteogenesis, angiogenesis, and reducing adipogenesis^24^, ^57^, which is full of expectations for the reconstruction of authentic bones.

Summary: The present study reports the promotion of OcD by activating the canonical Wnt signaling in isolated and cultured osteocytes, which is different from that in osteoblasts, from the perspective of cellular and molecular mechanisms. Osteocytic Wnt increases the TGFβ signaling, by which directly regulates RANKL transcription, and increases its expression to raise the ratio of RANKL/Opg for OcD and bone resorption. *In vivo* specific knockdown of osteocytic TGFβ signaling decreases RANKL expression and the generation and function of osteoclasts. This work reflects a classic case of how the cellular environment determines cell fate and provides scientists with a new perspective on how developmental signaling works. Osteoblasts are physiologically important for regulating bone metabolism and can also be used to rebuild metabolically functional bones.

### Online supplemental material

**Figure S1** shows daCO conditioned medium also contains higher concentration of Opg protein, but with higher RANKL/Opg ratio. **Figure S2** shows daCO increases the number of differentiated osteoclast precursors when co-cultured with BMM in growth medium, and enhanced OcD with OcD medium when co-cultured in non-contact Transwell assays. **Figure S3** shows the quality of the samples of wildtype osteocytes and daCO for RNA-seq assay. **Figure S4** shows the differentially regulated genes enriched from daCO transcriptome analysis that relate to osteoclast differentiation in the KEGG pathways. **Figure S5** shows Protein-Protein Interaction (PPI) network of the involved differentially regulated genes regarding osteoclast differentiation. **Figure S6** shows immunohistochemical staining for p-Smad2/3 in femoral longitudinal cross sections of mice with disrupted TGFβ signaling in osteoblasts. **Figure S7** shows the effect of osteoblastic Wnt on *Tgfb1/2* expression. **Figure S8** shows Wnt3a-CM upregulates RANKL/Opg and stimulates OcD in WTO. **Figure S9** shows significant elevation of RANKL and p-Smad2/3 in trabecular osteocytes of C91-treated mice. **Figure S10** shows low-dose TGFβ ligands stimulate p-Smad2/3, increase RANKL/Opg and enhance OcD in co-culture systems. **Table S1** lists the primers for qPCR (mouse). **Table S2** lists the si-RNA sequences for mouse RANKL. **Table S3** lists the ChIP-qPCR primers.

### Data availability

All data generated for this study is available from the corresponding authors upon reasonable request, and osteocyte sequencing data were deposited in the Gene Expression Omnibus (GEO) under accession number GSE301026.

## Supplementary Material

Supplementary figures and tables.

## References

[1] Seita J, Weissman I (2010). Hematopoietic stem cell: self-renewal versus differentiation. Wiley Interdiscip Rev Syst Biol Med.

[2] Teitelbaum SL (2000). Bone resorption by osteoclasts. Science (New York, NY).

[3] Charles JF, Aliprantis AO (2014). Osteoclasts: more than 'bone eaters'. Trends in molecular medicine.

[4] Okamoto K, Nakashima T, Shinohara M, Negishi-Koga T, Komatsu N, Terashima A (2017). Osteoimmunology: The Conceptual Framework Unifying the Immune and Skeletal Systems. Physiological reviews.

[5] Tsukasaki M, Takayanagi H (2019). Osteoimmunology: evolving concepts in bone-immune interactions in health and disease. Nature reviews Immunology.

[6] Asagiri M, Takayanagi H (2007). The molecular understanding of osteoclast differentiation. Bone.

[7] Nakashima T, Hayashi M, Fukunaga T, Kurata K, Oh-Hora M, Feng JQ (2011). Evidence for osteocyte regulation of bone homeostasis through RANKL expression. Nat Med.

[8] Xiong J, Onal M, Jilka RL, Weinstein RS, Manolagas SC, O'Brien CA (2011). Matrix-embedded cells control osteoclast formation. Nature medicine.

[9] Xiong J, Piemontese M, Onal M, Campbell J, Goellner JJ, Dusevich V (2015). Osteocytes, not Osteoblasts or Lining Cells, are the Main Source of the RANKL Required for Osteoclast Formation in Remodeling Bone. PLoS One.

[10] Veis DJ, O'Brien CA (2023). Osteoclasts, Master Sculptors of Bone. Annu Rev Pathol.

[11] Baron R, Kneissel M (2013). WNT signaling in bone homeostasis and disease: from human mutations to treatments. Nature medicine.

[12] Glass DA 2nd, Bialek P, Ahn JD, Starbuck M, Patel MS, Clevers H (2005). Canonical Wnt signaling in differentiated osteoblasts controls osteoclast differentiation. Developmental cell.

[13] Holmen SL, Zylstra CR, Mukherjee A, Sigler RE, Faugere MC, Bouxsein ML (2005). Essential role of beta-catenin in postnatal bone acquisition. The Journal of biological chemistry.

[14] Yamane T, Kunisada T, Tsukamoto H, Yamazaki H, Niwa H, Takada S (2001). Wnt signaling regulates hemopoiesis through stromal cells. Journal of immunology (Baltimore, Md: 1950).

[15] Qiang YW, Chen Y, Stephens O, Brown N, Chen B, Epstein J (2008). Myeloma-derived Dickkopf-1 disrupts Wnt-regulated osteoprotegerin and RANKL production by osteoblasts: a potential mechanism underlying osteolytic bone lesions in multiple myeloma. Blood.

[16] Fujita K, Janz S (2007). Attenuation of WNT signaling by DKK-1 and -2 regulates BMP2-induced osteoblast differentiation and expression of OPG, RANKL and M-CSF. Molecular cancer.

[17] Movérare-Skrtic S, Henning P, Liu X, Nagano K, Saito H, Börjesson AE (2014). Osteoblast-derived WNT16 represses osteoclastogenesis and prevents cortical bone fragility fractures. Nat Med.

[18] Yu B, Chang J, Liu Y, Li J, Kevork K, Al-Hezaimi K (2014). Wnt4 signaling prevents skeletal aging and inflammation by inhibiting nuclear factor-κB. Nat Med.

[19] Maeda K, Kobayashi Y, Udagawa N, Uehara S, Ishihara A, Mizoguchi T (2012). Wnt5a-Ror2 signaling between osteoblast-lineage cells and osteoclast precursors enhances osteoclastogenesis. Nat Med.

[20] Tu X, Delgado-Calle J, Condon KW, Maycas M, Zhang H, Carlesso N (2015). Osteocytes mediate the anabolic actions of canonical Wnt/β-catenin signaling in bone. Proceedings of the National Academy of Sciences of the United States of America.

[21] Osório J (2015). Bone. Osteocyte-specific activation of the canonical Wnt-β catenin pathway stimulates bone formation. Nature reviews Endocrinology.

[22] Buckland J (2015). Bone: Anabolic Wnt/β-catenin signalling: osteocytes are key. Nature reviews Rheumatology.

[23] Wang B, Khan S, Wang P, Wang X, Liu Y, Chen J (2022). A Highly Selective GSK-3β Inhibitor CHIR99021 Promotes Osteogenesis by Activating Canonical and Autophagy-Mediated Wnt Signaling. Front Endocrinol (Lausanne).

[24] Liu Y, Ruan X, Li J, Wang B, Chen J, Wang X (2022). The Osteocyte Stimulated by Wnt Agonist SKL2001 Is a Safe Osteogenic Niche Improving Bioactivities in a Polycaprolactone and Cell Integrated 3D Module. Cells.

[25] Bivi N, Condon KW, Allen MR, Farlow N, Passeri G, Brun LR (2012). Cell autonomous requirement of connexin 43 for osteocyte survival: consequences for endocortical resorption and periosteal bone formation. J Bone Miner Res.

[26] Harada N, Tamai Y, Ishikawa T, Sauer B, Takaku K, Oshima M (1999). Intestinal polyposis in mice with a dominant stable mutation of the beta-catenin gene. The EMBO journal.

[27] Qiao X, Wu X, Zhao Y, Yang Y, Zhang L, Cai X (2023). Cell Transitions Contribute to Glucocorticoid-Induced Bone Loss. Cells.

[28] Dole NS, Mazur CM, Acevedo C, Lopez JP, Monteiro DA, Fowler TW (2017). Osteocyte-Intrinsic TGF-β Signaling Regulates Bone Quality through Perilacunar/Canalicular Remodeling. Cell reports.

[29] Otero K, Shinohara M, Zhao H, Cella M, Gilfillan S, Colucci A (2012). TREM2 and beta-catenin regulate bone homeostasis by controlling the rate of osteoclastogenesis. J Immunol.

[30] Wang X, Ma Y, Chen J, Liu Y, Liu G, Wang P (2023). A novel decellularized matrix of Wnt signaling-activated osteocytes accelerates the repair of critical-sized parietal bone defects with osteoclastogenesis, angiogenesis, and neurogenesis. Bioact Mater.

[31] Zhao S, Zhang YK, Harris S, Ahuja SS, Bonewald LF (2002). MLO-Y4 osteocyte-like cells support osteoclast formation and activation. Journal of bone and mineral research: the official journal of the American Society for Bone and Mineral Research.

[32] Itonaga I, Sabokbar A, Neale SD, Athanasou NA (1999). 1,25-Dihydroxyvitamin D(3) and prostaglandin E(2) act directly on circulating human osteoclast precursors. Biochemical and biophysical research communications.

[33] Vesprey A, Yang W (2016). Pit Assay to Measure the Bone Resorptive Activity of Bone Marrow-derived Osteoclasts. Bio-protocol.

[34] Tu X, Rhee Y, Condon KW, Bivi N, Allen MR, Dwyer D (2012). Sost downregulation and local Wnt signaling are required for the osteogenic response to mechanical loading. Bone.

[35] McCann J, Sosa-Miranda CD, Guo H, Reshke R, Savard A, Zardini Buzatto A (2022). Contaminating transfection complexes can masquerade as small extracellular vesicles and impair their delivery of RNA. Journal of extracellular vesicles.

[36] Jiang Y, Li F, Gao B, Ma M, Chen M, Wu Y (2021). KDM6B-mediated histone demethylation of LDHA promotes lung metastasis of osteosarcoma. Theranostics.

[37] Hilton MJ, Tu X, Wu X, Bai S, Zhao H, Kobayashi T (2008). Notch signaling maintains bone marrow mesenchymal progenitors by suppressing osteoblast differentiation. Nat Med.

[38] Dempster DW, Compston JE, Drezner MK, Glorieux FH, Kanis JA, Malluche H (2013). Standardized nomenclature, symbols, and units for bone histomorphometry: a 2012 update of the report of the ASBMR Histomorphometry Nomenclature Committee. Journal of bone and mineral research: the official journal of the American Society for Bone and Mineral Research.

[39] Griffin JN, Del Viso F, Duncan AR, Robson A, Hwang W, Kulkarni S (2018). RAPGEF5 Regulates Nuclear Translocation of β-Catenin. Dev Cell.

[40] Wilson SR, Peters C, Saftig P, Brömme D (2009). Cathepsin K activity-dependent regulation of osteoclast actin ring formation and bone resorption. The Journal of biological chemistry.

[41] Sun K, Zhu J, Deng Y, Xu X, Kong F, Sun X (2021). Gamabufotalin Inhibits Osteoclastgenesis and Counteracts Estrogen-Deficient Bone Loss in Mice by Suppressing RANKL-Induced NF-κB and ERK/MAPK Pathways. Frontiers in pharmacology.

[42] Inoue M, Nagai-Yoshioka Y, Yamasaki R, Kawamoto T, Nishihara T, Ariyoshi W (2022). Mechanisms involved in suppression of osteoclast supportive activity by transforming growth factor-β1 via the ubiquitin-proteasome system. PLoS One.

[43] Kudipudi PK, Galuska SP, Dietze R, Scheiner-Bobis G, Loveland KL, Konrad L (2019). Betaglycan (TβRIII) is a Key Factor in TGF-β2 Signaling in Prepubertal Rat Sertoli Cells. Int J Mol Sci.

[44] Willems E, Cabral-Teixeira J, Schade D, Cai W, Reeves P, Bushway PJ (2012). Small molecule-mediated TGF-β type II receptor degradation promotes cardiomyogenesis in embryonic stem cells. Cell stem cell.

[45] Kim N, Kadono Y, Takami M, Lee J, Lee SH, Okada F (2005). Osteoclast differentiation independent of the TRANCE-RANK-TRAF6 axis. J Exp Med.

[46] Xia Y, Inoue K, Du Y, Baker SJ, Reddy EP, Greenblatt MB (2022). TGFβ reprograms TNF stimulation of macrophages towards a non-canonical pathway driving inflammatory osteoclastogenesis. Nat Commun.

[47] Kramer I, Halleux C, Keller H, Pegurri M, Gooi JH, Weber PB (2010). Osteocyte Wnt/beta-catenin signaling is required for normal bone homeostasis. Mol Cell Biol.

[48] Scheven BA, Kawilarang-De Haas EW, Wassenaar AM, Nijweide PJ (1986). Differentiation kinetics of osteoclasts in the periosteum of embryonic bones *in vivo* and *in vitro*. The Anatomical record.

[49] Jacome-Galarza CE, Percin GI, Muller JT, Mass E, Lazarov T, Eitler J (2019). Developmental origin, functional maintenance and genetic rescue of osteoclasts. Nature.

[50] Kode A ea (2014). Leukaemogenesis induced by an activating β-catenin mutation in osteoblasts. Nature.

[51] Wang P, Wang X, Wang B, Li X, Xie Z, Chen J (2022). 3D printing of osteocytic Dll4 integrated with PCL for cell fate determination towards osteoblasts *in vitro*. Bio-Design and Manufacturing.

[52] Zhang Y, Zhao Y, Xie Z, Li M, Liu Y, Tu X (2022). Activating Wnt/β-Catenin Signaling in Osteocytes Promotes Osteogenic Differentiation of BMSCs through BMP-7. Int J Mol Sci.

[53] Han Y, You X, Xing W, Zhang Z, Zou W (2018). Paracrine and endocrine actions of bone-the functions of secretory proteins from osteoblasts, osteocytes, and osteoclasts. Bone research.

[54] Hsiao CY, Chen TH, Chu TH, Ting YN, Tsai PJ, Shyu JF (2020). Calcitonin Induces Bone Formation by Increasing Expression of Wnt10b in Osteoclasts in Ovariectomy-Induced Osteoporotic Rats. Frontiers in endocrinology.

[55] Zheng CM, Hsu YH, Wu CC, Lu CL, Liu WC, Zheng JQ (2019). Osteoclast-Released Wnt-10b Underlies Cinacalcet Related Bone Improvement in Chronic Kidney Disease. Int J Mol Sci.

[56] Black DM, Geiger EJ, Eastell R, Vittinghoff E, Li BH, Ryan DS (2020). Atypical Femur Fracture Risk versus Fragility Fracture Prevention with Bisphosphonates. The New England journal of medicine.

[57] Zhang J, Zhang Y, Chen J, Gong W, Tu X (2024). The Osteocyte with SB216763-Activated Canonical Wnt Signaling Constructs a Multifunctional 4D Intelligent Osteogenic Module. Biomolecules.

