# Supplementary material for: Osteocytes Produces RANKL Via Wnt-TGFβ Signaling Axis for Osteoclastogenesis
Source: Int J Biol Sci. 2025 Sep 12;21(13):5821–41. doi: 10.7150/ijbs.117481 (PMC12509901; doi:10.7150/ijbs.117481)
Supplement: Supplementary file 1 — Supplementary figures and tables. [file ijbsv21p5821s1.pdf]

Osteocytic Wnt-TGFβ signaling transduction axis activates RANKL transcription for osteoclastogenesis

Yujiao Liu[1,2, #], Lizhou Zhao[1, #], Molin Li[1, #], Weimin Gong[1], Xiaofang Wang[1], Ying Zhang[1], Pengtao Wang[1], Yisheng Luo[1], Yining Zhang[1], Yufei Shao[3], Makoto Mark Taketo[4], Teresita Bellido[5], Gaohai Shao[2], Xing Liu[3, ✉], and Xiaolin Tu[1,6, ✉]

[1] Laboratory of Skeletal Development and Regeneration, Key Laboratory of Clinical Laboratory Diagnostics (Ministry of Education), College of Laboratory Medicine, Chongqing Medical University, Chongqing 400016, China

[2] Department of Orthopedics, Affiliated Yongchuan Hospital of Chongqing Medical University, Chongqing 402160, China

[3] Department of Orthopedics, Ministry of Education Key Laboratory of Child Development and Disorders, National Clinical Research Center for Child Health and Disorders, Children's Hospital of Chongqing Medical University, Chongqing, 400014, China

[4] Department of Pharmacology, Graduate School of Medicine, Kyoto University, Kyoto, 606-8501, Japan

[5] Department of Physiology and Cell Biology, University of Arkansas for Medical Sciences, Little Rock, AR 72223, USA

[6] Department of Anatomy, Cell Biology and Physiology, Indiana University School of Medicine, Indianapolis, IN, 46202, USA

[#] YJ.L., LZ.Z., and ML.L. these authors contributed equally to this work

✉ Correspondence: xtu@cqmu.edu.cn, Tel: +86-185-2382-0685; liuxinda@163.com, Tel: +86-136-3780-8322.

Supplementary information

Supplementary information, Figure. S1

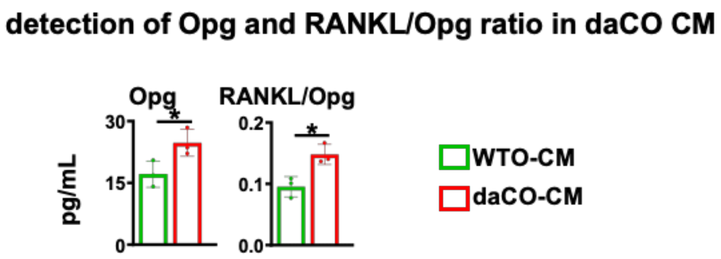

Supplementary information, Fig. S1 daCO conditioned medium has higher concentration of Opg protein with higher RANKL/Opg ratio. Opg protein concentration in osteocyte culture medium as detected by ELISA. Data were expressed as mean ± SD. WTO, wild-type osteocytes; daCO, osteocytes with dominantly active β-catenin. *p < 0.05 v.s. WTO-CM by t-test, n=3.

Supplementary information, Figure. S2

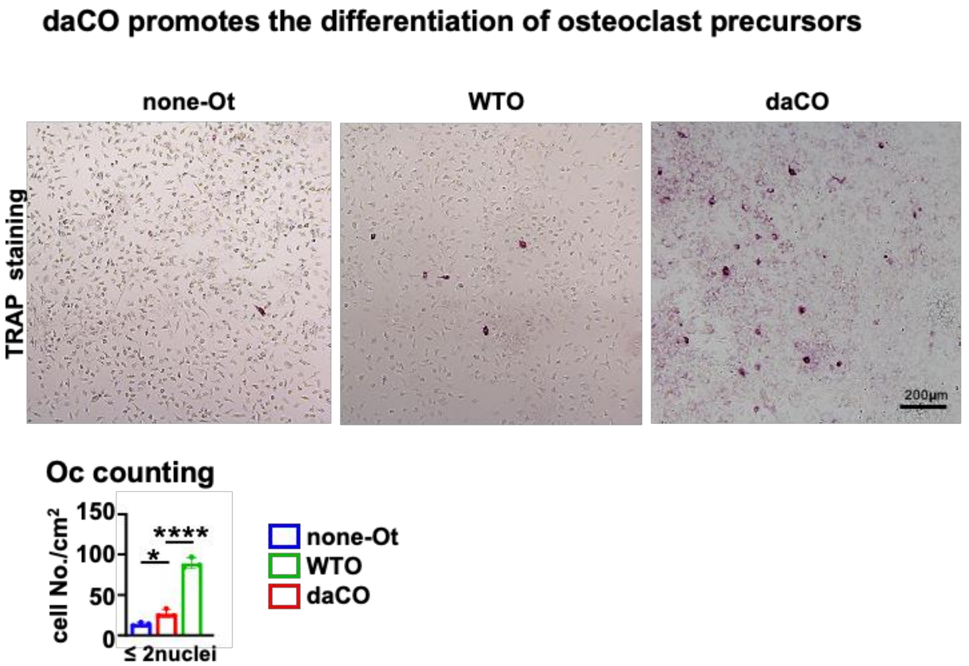

Supplementary information, Fig. S2 daCO induces the differentiation of osteoclast precursors in growth medium. TRAP staining of the co-culture of BMMs and osteocytes in growth medium for 11 days and TRAP-positive cell counting. Data were expressed as mean ± SD. *$p < 0.05$ v.s. only BMMs without osteocytes, $^{\#}$ $p < 0.05$ v.s. WTO by One-Way ANOVA, n=3.

Supplementary information, Figure. S3

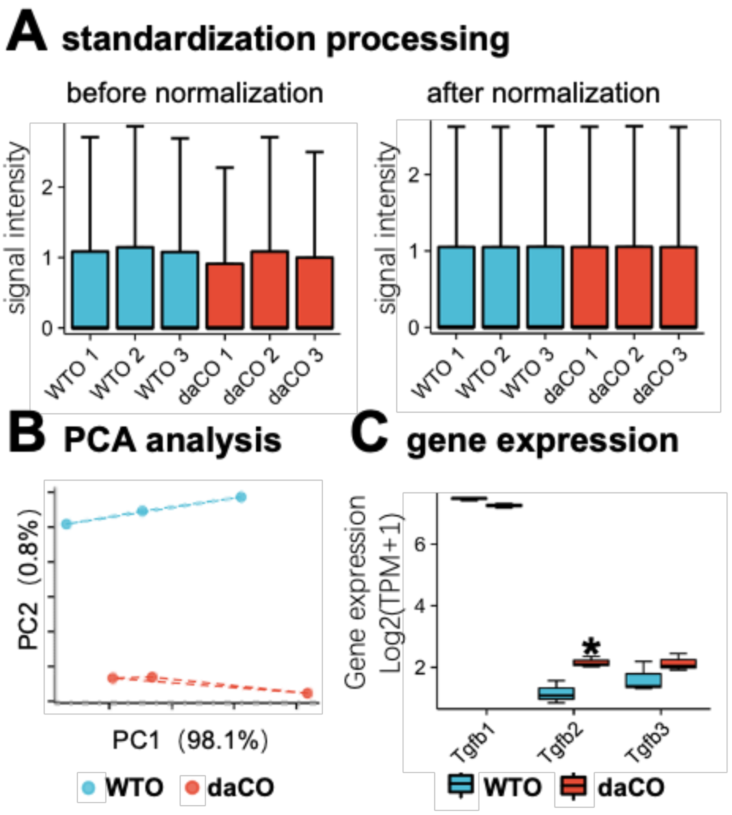

Supplementary information, Figure. S3 Samples of WTO and daCO for RNA-seq assay. A The baseline levels of raw detected signal before and after standardized using log2+1 normalization. **B** Analyze significant differences and separation between the daCO and WTO groups using PCA dimensionality reduction. **C** The expression of *Tgfb1*, *Tgfb2*, and *Tgfb3* using grouped box plots. PCA, Principal Component Analysis.

Supplementary information, Figure. S4

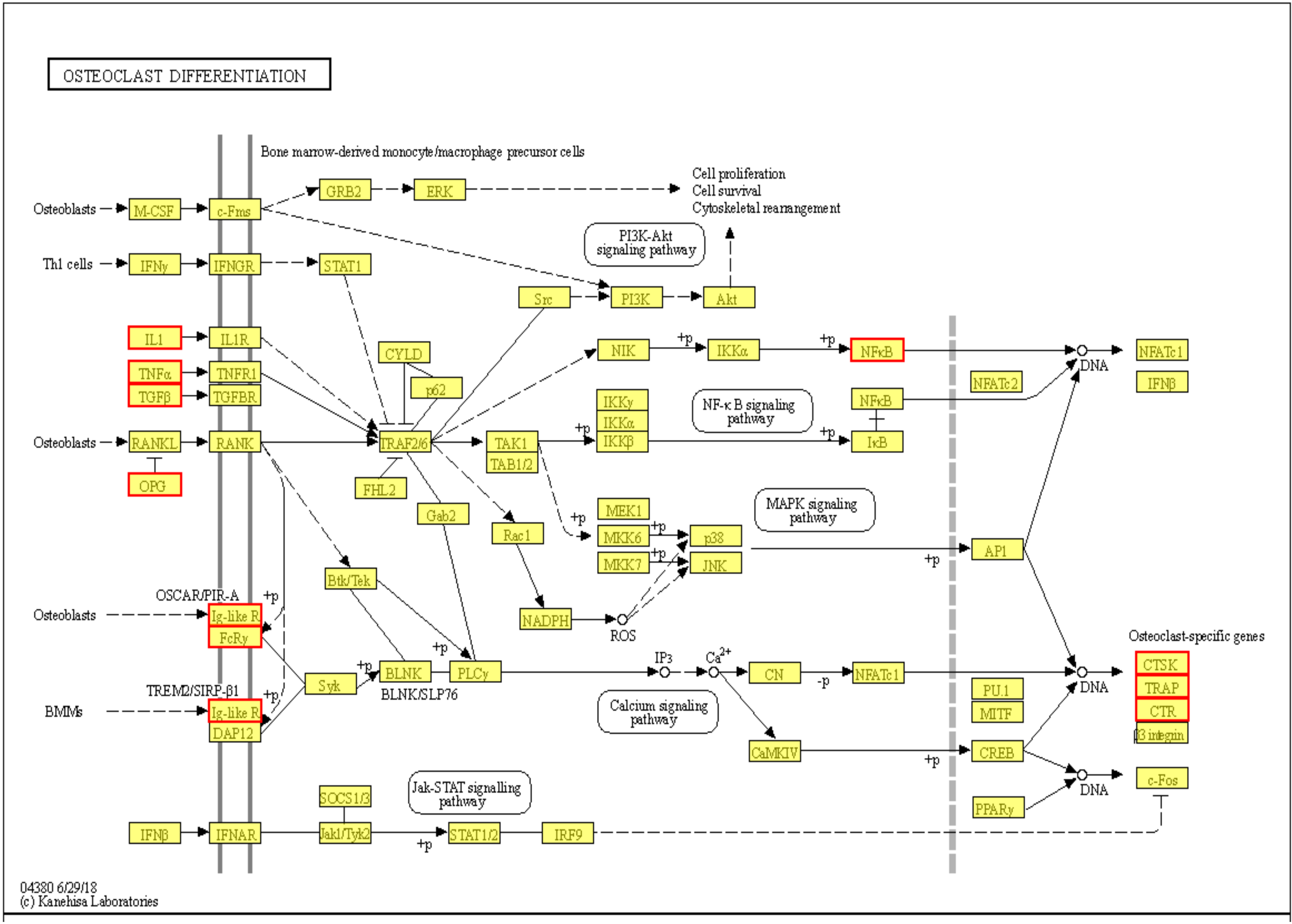

Supplementary information, Figure. S4 The KEGG pathways of osteoclast differentiation involves the differentiated expressed genes enriched from daCO transcriptome analysis that are most correlated with osteocytic Wnt. Osteoclast differentiation includes IL-1, TNFα, TGFβ, Opg, Ig-like R/FcRy pathways in the upstream of osteoclast differentiation and its downstream, such as NFκB, CTSK, TRAP, calcitonin receptor.

Supplementary information, Figure. S5

PPI analysis for the proteins related to the differentially expressed OcD genes in daCO

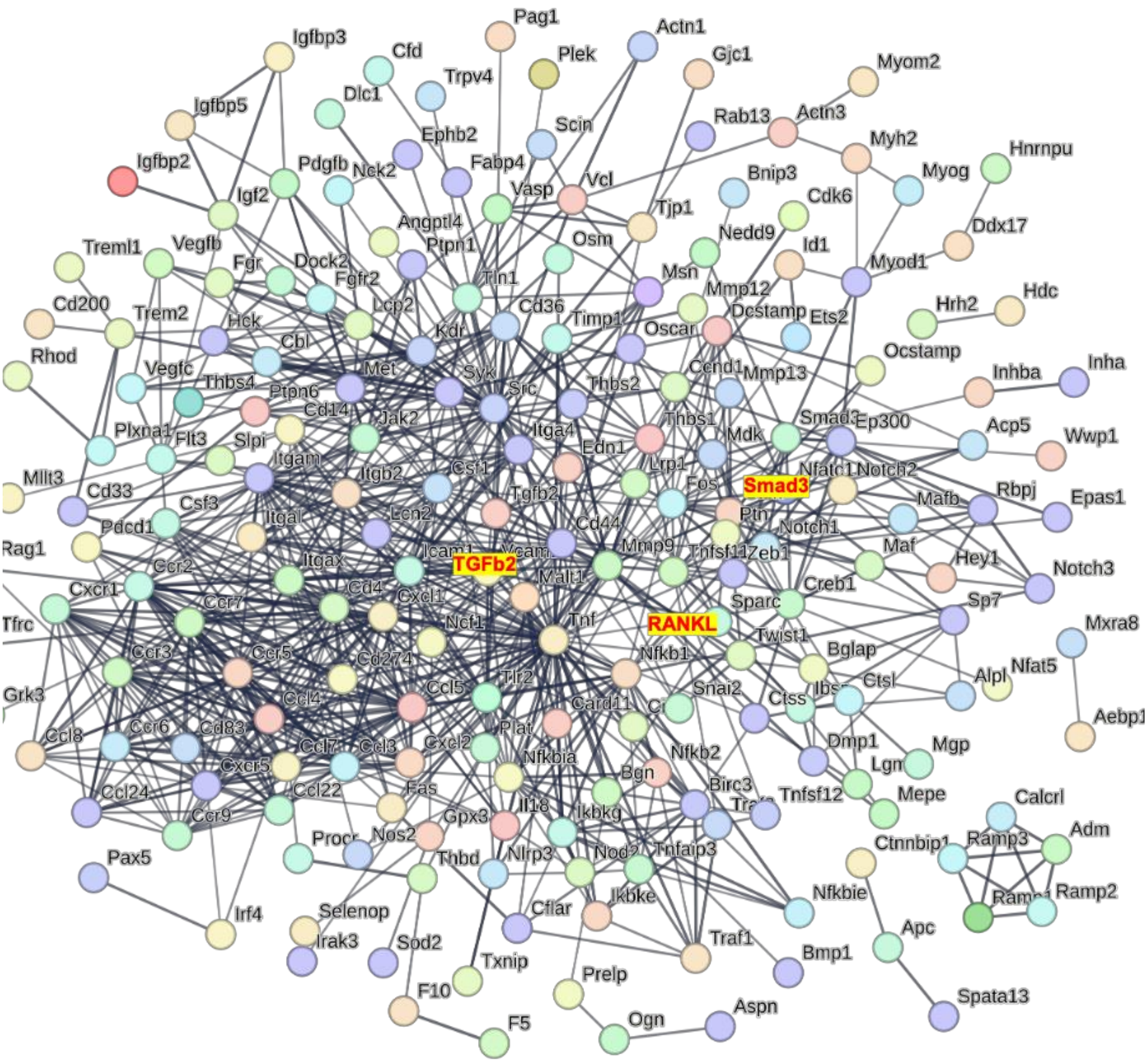

Supplementary information, Figure. S5 Protein-Protein Interaction (PPI) network of the involved differentially regulated genes regarding OcD.

Supplementary information, Figure. S6

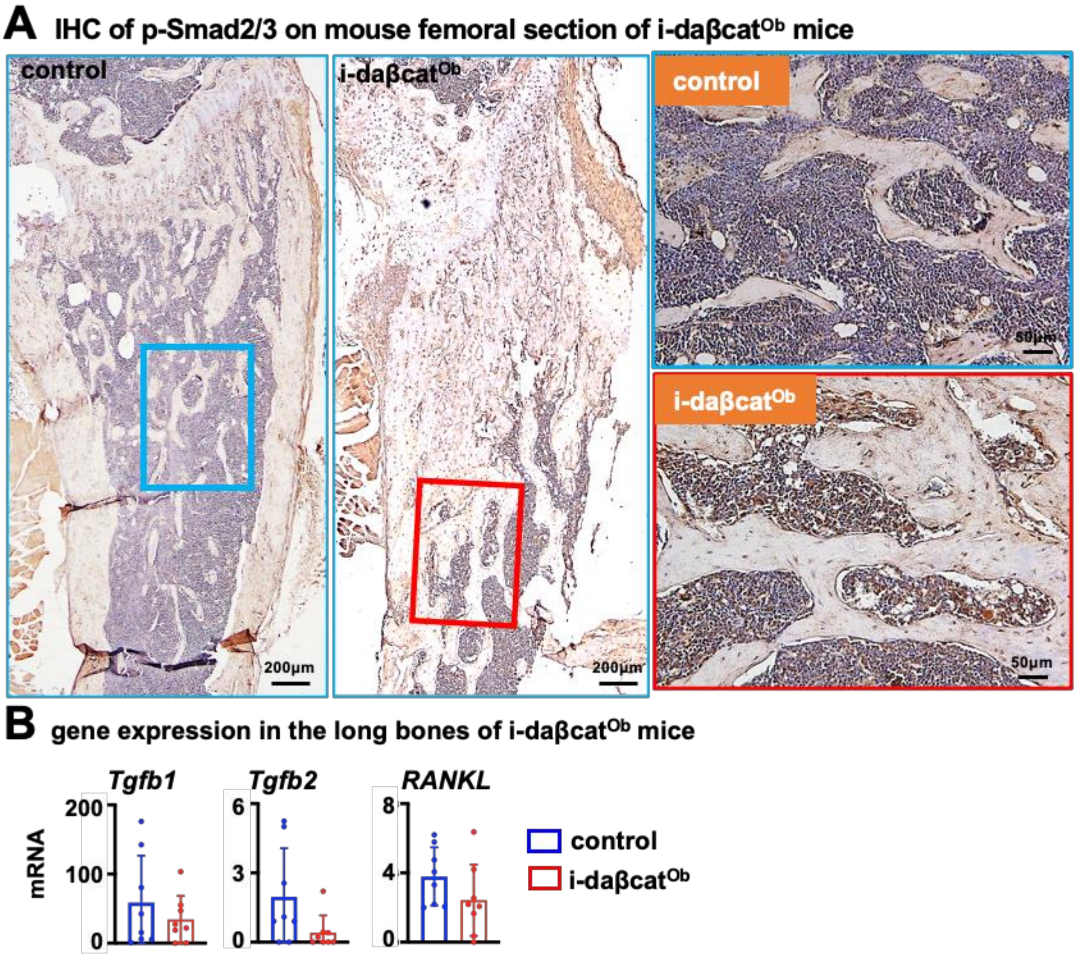

Supplementary information, Figure. S6 The effect of osteoblastic Wnt on TGFβ signaling. A Tgfb1, Tgfb2, and RANKL expression of the femurs of i-daβcat^{Ob} mice. **B** Immunohistochemical analysis of the p-Smad2/3 of the TGF-β signaling pathway in the longitudinal cross sections of mouse femurs. *$p < 0.05$ v.s. control mice by *t*-test, n=8. i-daβcat^{Ob}, tamoxifen-induced expression of dominantly active β-catenin in osteoblasts.

Supplementary information, Figure. S7

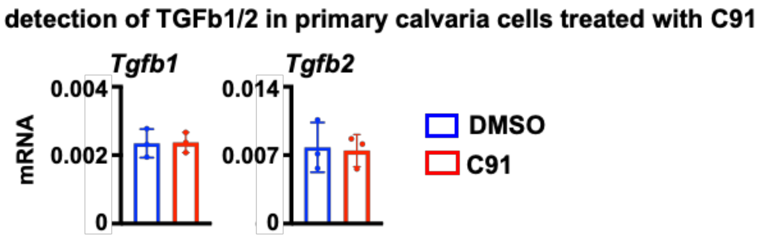

Supplementary information, Figure. S7 The effect of osteoblastic Wnt on TGFβ signaling. Tgfb1 and Tgfb2 expression of the mouse primary calvaria cells treated with Wnt agonist C91. n=3.

Supplementary information, Table. S1

Table S1. Sequences of primers for RT-PCR (mouse)

Primer	Forward	Reverse
Lef1	TACCCCAGCCAGTGTCAACA	TCCATGATAGGCTTTGATGACTTTC
Axin2	TGCAGGAGGCGGTACAGTTC	GCTGGAAGTGGTAAAGCAGCTT
Bmp4	GAGGAGTTTCCATCACGAAGA	GCTCTGCCGAGGAGATCA
Smad6	AAGATGCTGAAGCCGTTGGT	CGAACTCCAGTATCTCCGCTTT
Alpl	ACACCAATGTAGCCAAGAATGTCA	GATTCGGGCAGCGGTTACT
Col1a1	GCTGGCAAGAATGGCGAC	AAGCCACGATGACCCTTTATG
Sost	CGTGCCTCATCTGCCTACTTGTG	CCGGTTCATGGTCTGGTTGTTCTC
Dmp-1	CATTCTCCTTGTGTTCCTTTGG	TCAGTATTGTGGTATCTGGCAACT
RANKL	CATGACGTTAAGCAACGG	AGGGAAGGGTTGGACA
Opg	ACGGACAGCTGGCACACCAG	CTCACACACTCGGTTGTGGG
Rank	TGAGCCTCCGAGCAGAACTGAC	CTGCCTGTGTAGCCATCTGTTGAG
MCSF	CGCTGCCCRTCTTCGACAT	TCTGACACCTCCTTGGCAATACT
Ctsk	GGCAGGGTCCCAGACTCCATC	TGAAAGCCCAACAGGAACCACAC
Dcstamp	TCCTCCATGAACAAACAGTTCCAA	AGACGTGGTTTAGGAATGCAGCTC
Nfatc1	GAGACAGACATCGGGAGGAAGA	GTGGGATGTGAACTCGGAAGA
CHoB	CAGAATGGTAGGAAGGTCACG	CGAATGCTGTAATGGCGTATC

Supplementary information, Table. S2

Table S2. si-RNA sequence

Gene name	Sequence
RANKL-siRNA1	GGATGAAACAAGCCTTTCA
RANKL-siRNA2	CAGACTATCTTCAGCTGAT
RANKL-siRNA3	CATGACGTTAAGCAACGGA

Supplementary information, Table. S3

Table S3. ChIP-qPCR primer sequences designed for 5 peaks

Primer	Forward	Reverse
P1	GAGGAGACCACCATCAAGAATCGT	TTGTCTAGTTCTTCCTGGCTCACC
P2-1	AGGTGAGGTGCTGTCCCTTAGAA	CCTGTGGAAGTGTCTGTTAGAGAACT
P2-2	GTCTTGAATCAGAATTGTGCCTTTAGG	GGGACAGCACCTCACCTTTGAA
P2-3	CCTCAATGCTGGGATTAAAGGTATGT	GTCACCTAAAGGCACAATTCTGATTC
P3-1	CGTTTCTCCCATCCAAGTGCTAACC	GCCTCTATCCTGGTGGTGATCTGAT
P3-2	GAACCATCAGATCACCACCAGGATAG	ACAGAGTTGAGTACATACTGACCTTGG
P4-1	AGGCGTCCAACTATGTAGACTGAAC	ACAGGAGCAGAACCGATAGAATGAA
P4-2	CCACCTTACACTGCTCGGTTCATT	TCAGTCTACATAGTTGGACGCCTCA
P4-3	TTCATTCTATCGGTTCTGCTCCTGT	GCCAAGAGTTCTGCCTGAGTCTC
P5-1	CTCTAATCCACAGCCTTCCTGACTG	CCTGCTTCCTGACCTGCTTGAG
P5-2	TCAAGCAGGTCAGGAAGCAGGAG	GCTGATGACACAAAGGTGAGGAAGT
